# Metformin for primary prevention of colorectal neoplasms in adenoma-free populations: a systematic review and dose-response meta-analysis

**DOI:** 10.3389/fphar.2025.1645387

**Published:** 2025-11-19

**Authors:** Mengdan Shen, Shan Lu, Zihao Xu, Feifei Zhou, Li-Ting Sheng, Qiang Yu

**Affiliations:** 1 Department of Gastroenterology, The Affiliated Suzhou Hospital of Nanjing Medical University, Suzhou Municipal Hospital, Gusu School, Nanjing Medical University, Suzhou, Jiangsu, China; 2 Wuxi Second Geriatric Hospital, Wuxi Huishan Second People’s Hospital, Wuxi, Jiangsu, China; 3 Department of Gastroenterology, Chengnan Street Community Health Service, Suzhou, Jiangsu, China; 4 Phase I Clinical Trial Center, The Affiliated Suzhou Hospital of Nanjing Medical University, Suzhou Municipal Hospital, Gusu School, Nanjing Medical University, Suzhou, Jiangsu, China

**Keywords:** metformin, colorectal neoplasm, cancer, adenoma, primary prevention

## Abstract

**Background:**

Metformin shows promise in preventing colorectal cancer (CRC) and its precursors, but evidence on its dose-response effect remains limited.

**Aim:**

To determine the association between metformin therapy and colorectal neoplasms in adenoma-free individuals and characterize the dose-response relationship.

**Methods:**

Adjusted effect estimates from each study were aggregated using a random-effect model. Subgroup analyses, publication bias assessment, sensitivity analyses and dose-response analyses were conducted.

**Results:**

A total of 37 eligible studies, involving 1,416,085 participants, were included. Metformin significantly reduced colorectal neoplasms risk (Hazard ratio (HR) = 0.79, 95% confidence interval (CI), 0.73–0.85, Odds ratio (OR) = 0.80, 95% confidence interval, 0.74–0.87). Subgroup analyses demonstrated enhanced efficacy in Asian populations, younger patients (<60 years), and cohorts with ≥50% male participants. Dose-response analysis identified 331 mg/day as the optimal dose for CRC risk reduction (OR = 0.83, 95% CI, 0.76–0.91). Each additional year of use reduced CRC risk by 3% (OR = 0.97, 95% CI, 0.95–0.99).

**Conclusion:**

Metformin demonstrates effective chemoprevention against colorectal neoplasms, where the inverse association was most prominent at low-dose, long-term therapy.

**Systematic Review Registration:**

https://www.crd.york.ac.uk/prospero/, Identifier CRD42023394042.

## Introduction

Colorectal cancer (CRC) is a prevalent malignant tumor with rising global incidence and mortality rates. According to the latest global cancer incidence and mortality estimates, CRC is the third major cause of cancer and the second leading cause of cancer-related deaths worldwide (Bray et al., 2024). Colorectal adenoma (CRA), commonly denoted as the precursor lesion of CRC, plays an indispensable role in the progression process in the development of CRC. A classic “normal mucosa - CRAs - CRC” progression pathway is considered the primary process in the evolution of CRC (Vogelstein et al., 1988). Currently, the treatment of CRA and CRC relies on endoscopic and surgical resection, systemic adjuvant chemotherapy, radiation therapy, targeted therapy, and immunotherapy. With advances in treatment methods and medical devices, 5-year relative survival rate for CRC has increased (Siegel et al., 2023). However, the disease burden such as soreness, adverse effects, poor patient compliance and high therapy costs continue to persist. In addition, 25%–50% of early-stage CRC has the potential to progress to metastasis over time (Fan et al., 2021). Therefore, early prevention of colorectal neoplasms is of critical importance for public health.

Epidemiological and biological evidence reported that individuals with diabetes mellitus (DM) have an increased risk of developing several types of cancers, especially digestive system cancers represented by CRC (Fernandez et al., 2021; Lawler et al., 2023; Peeters et al., 2015; Johnson et al., 2012). In a recent cohort study involving 54,597 adults, the investigators reported a 47% increased risk of CRC risk compared with non-diabetic individuals (Lawler et al., 2023). Consequently, individuals with diabetes should be considered a priority population for colorectal cancer prevention efforts. Metformin is considered as the first-line treatment option for the management of non-insulin dependent DM, earning its inclusion on the World Health Organization’s list of essential medicines since 2019 (Davies et al., 2018; World Health, 2019). Metformin is highly appealing as a target for antitumor research and exerts its anticancer effects through multiple mechanisms, including metabolism, epigenetics, cell cycle, migration, metastasis, cell death, cell senescence, cancer stem cells, immunity, and gut microbes (Hua et al., 2023). The central mechanism of metformin’s anticancer activity is the regulation of energy metabolism, with the key pathway being the activation of the adenosine monophosphate-activated protein kinase (AMPK)/mammalian target of rapamycin (mTOR) pathway triggered by inhibition of complex I in the mitochondrial respiratory chain (Zhou et al., 2001; Spiering, 2019; Lee et al., 2011). Therefore, metformin holds promise as a potential candidate for chemoprevention, particularly in reducing colorectal cancer morbidity among individuals with diabetes.

A number of studies have explored the connection between metformin use and colorectal cancer but have yielded contradictory results. Several large-scale, population-based observational epidemiologic studies have reported strong inverse associations between metformin use and risk of colorectal cancer (Lee et al., 2011; Rosato et al., 2016; Seo et al., 2022; Tseng, 2017; Libby et al., 2009). However, a subset of researchers have argued that the protective effect of metformin may be overestimated (Suissa and Azoulay, 2012; Tsilidis et al., 2014). Higher-quality clinical trials have consistently demonstrated comparable findings. Higurashi et al. demonstrated the anti-tumor effects of metformin, while studies by Zell and Park et al. showed no significant effect (Zell et al., 2020; Park et al., 2021; Higurashi et al., 2016). Furthermore, previous studies have predominantly focused on the outcome of CRC while neglecting CRA. Adenomas often used in studies as surrogate marker to reduce the need for long follow-up or larger sample sizes (Levin, 2003). Given that most cases of CRC develop by the way of “normal mucosa - CRAs - CRC” progression, whether metformin plays a key role in the stage of precancerous lesions is essential for developing effective chemopreventive strategies. In addition, there is still no comprehensive and quantitative evaluation of published research on this topic.

To overcome these limitations, we conducted a comprehensive systematic review and meta-analysis to consolidate the existing evidence on the association between metformin use and colorectal neoplasms, considering both CRC and CRA outcomes, and to clarify the dose-response relationship underlying its chemopreventive effects.

## Methods

This meta-analysis was conducted according to the guidelines of Preferred Reporting Items for Systematic Reviews and Meta-Analyses (PRISMA) (Matthew et al., 2021) and Meta-analysis of Observational Studies in Epidemiology recommendations (Stroup et al., 2000) and was registered at the International Prospective Register of Systematic Reviews with the registration number CRD42023394042.

### Data sources and searches

We conducted an initial search of electronic databases through PubMed and Embase on 11 November 2022. A subsequent search update was performed on 20 March 2024, utilizing the identical databases for consistency. Our search employed targeted keywords pertinent to “metformin,” “colorectal cancer,” and “colorectal adenoma,” with the specific methodologies detailed in Supplementary Table S2. Titles and abstracts were screened to eliminate irrelevant studies, followed by a full-text review to evaluate eligible articles. The reference lists of each primary eligible study, along with previous systematic reviews, were scanned to identify additional relevant studies. Each stage of study selection, data extraction, and study quality assessment was independently conducted by the same two investigators (Mengdan, Shen and Shan, Lu). Any discrepancies in the above process finally reached a consensus through a negotiation conference with a third reviewer (Li-Ting, Sheng).

### Inclusion and exclusion criteria

Studies were included if they met the following criteria: (1) observational studies (cohort, case–control or nested case–control) or randomized controlled trials; (2) studies that exposure to any dose or duration of metformin with no use of metformin or other anti-diabetic drugs for comparison; (3) studies that listed occurrence of incident colorectal adenoma and colorectal cancer as an outcome of interest; (4) studies that reported the risk point estimate as an adjusted odds ratio (OR), hazard ratio (HR), or relative risk (RR) with a 95% confidence interval (CI); (5) articles written or translated into the English language. Studies were excluded if they (1) enrolled participants with a prior history of colorectal adenomas; (2) did not report specific results or contained duplicate or identical data; or (3) were case reports, systematic reviews, meta-analyses, editorials, commentaries, guidelines, conference abstracts, or letters. For the included studies, prior adenoma status was generally based on the absence of a documented diagnosis of colorectal adenomas or colorectal cancer.

### Data extraction and quality assessment

We designed standardized forms to extract data on the first author, year of the publication, country of study location, study type, total number of participants, comparison and demographic features such as sex, age and body mass index (BMI). Adjusted effect estimates with 95% CI as well as covariates that were adjusted in the multivariable analysis were recorded.

The Newcastle–Ottawa Scale (NOS) was utilized to evaluate the methodological quality of observational studies. It consists of three dimensions (group selection, groups comparability and exposure), eight items and a maximum score of 9. Scores of 7–9, 4–6 and 0–3 were categorized as high, moderate and low quality, respectively.

### Statistical methods

All statistical analyses were performed using Stata/SE 16.0 software and p ≤ 0.05 was considered as a statistically significant result. The association between metformin therapy and colorectal neoplasms was assessed by consolidating the point estimate from each study using the generic inverse variance method of DerSimonian and Laird. Results stratified by gender were regarded as two distinct studies. Due to the relatively infrequent outcome, we assumed that the reported RRs were approximately ORs to simplify the results and ensure the comprehensiveness of the analysis and maximization of the statistical power (Greenland, 1987). Q test and I^2^ statistics were employed to evaluate the extent of heterogeneity among the studies. An I^2^ value of 0%–25% denotes heterogeneity might not be important, 26%–50% denotes low heterogeneity, 51%–75% denotes moderate heterogeneity, and more than 75% denotes high heterogeneity (Higgins et al., 2003). A random-effects model was employed if significant heterogeneity (I^2^ statistic >50% or Q test <0.1) was observed (DerSimonian and Laird, 2015). Subgroup analysis was conducted by categorizing the studies based on outcome (cancer or adenoma), region (America, Europe, or Asia), mean age (<60 years, 60–70 years, or ≥70 years), male percentage (≥50%, <50%) and mean BMI (24–28 kg/m^2^, ≥28 kg/m^2^). Publication bias was assessed by funnel plots as well as Egger’s test. Sensitivity analyses were performed through restricting the analytical cohort to studies meeting the following prespecified criteria: (1) cohort study design; (2) high methodological quality (NOS score ≥7); (3) comparator groups utilizing other antidiabetic medications (4) study populations exclusively comprising individuals with diabetes mellitus.

Further assessment of dose-response relationship between metformin use and CRC risk was conducted under the generalized least squares trend (GLST) estimation and restricted cubic spline random-effects model with four knots at the 5%, 35%, 65% and 95% percentile (Orsini et al., 2006; Orsini and Greenland, 2011). To verify the model, a p value for non-linear relationship was computed by testing the null hypothesis that the coefficient of the second spline equaled zero. If model verification indicated significance (p < 0.05), a non-linear model was adopted; otherwise, a linear model was used. Reported doses were consistently converted to the unit milligram/day. Since some studies did not report the number of cases in each category, we estimated these data based on the number of total cases and the reported effect size (Bekkering et al., 2008). When exposures were reported as a range, we took the midpoint. For open-ended categories, we set the dose to be 20% higher or lower than the nearest breakpoint, which proved to produce a better fit (Berlin et al., 1993; Filippini et al., 2022).

## Results

### Search results and characteristics

**FIGURE 1:**
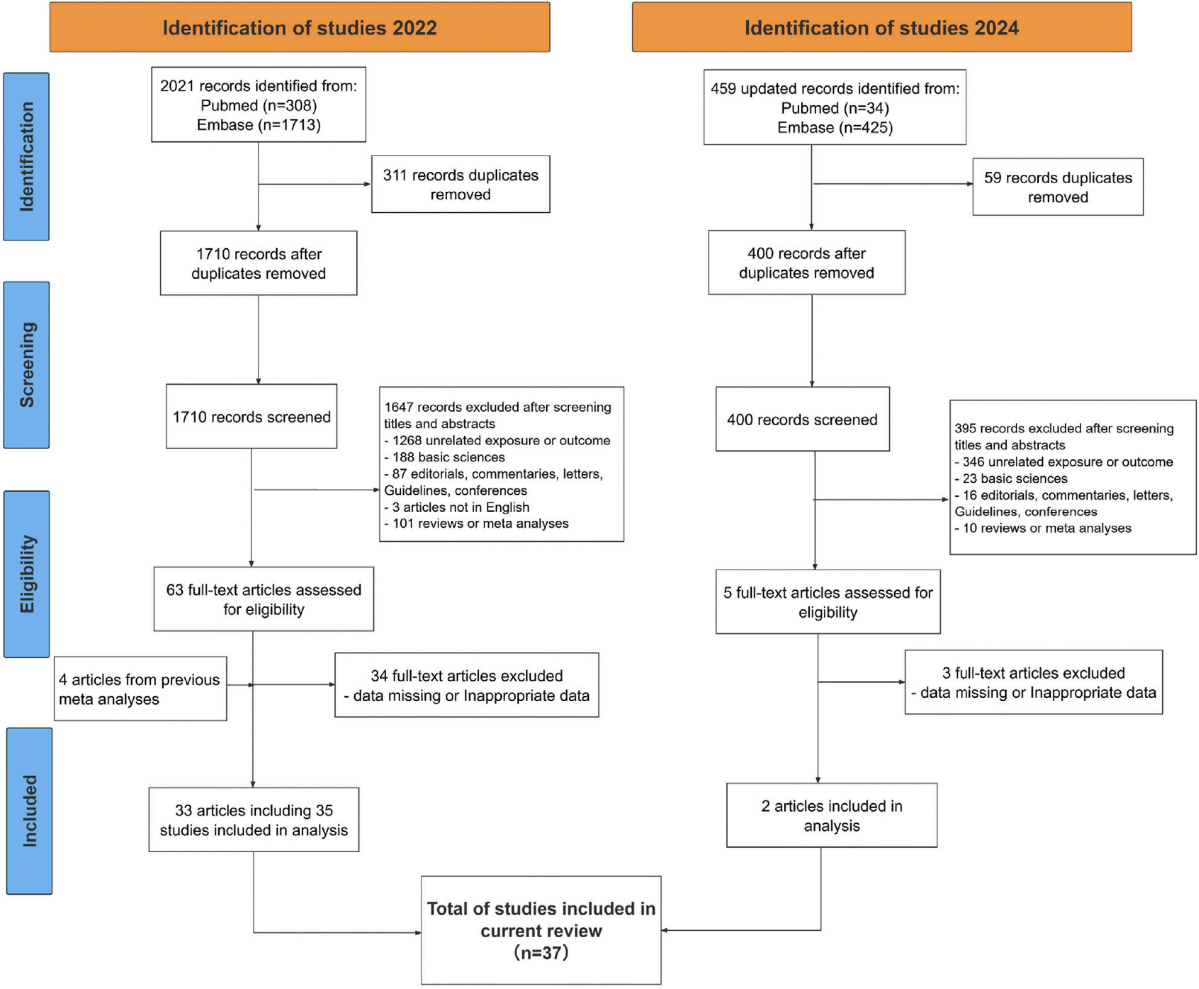
Preferred Reporting Items for Systematic Reviews and Meta-Analyses (PRISMA) diagram of included studies.

During the initial search, we identified 2021 results from Embase and PubMed and then removed 311 duplicated records and 1647 records based on titles and abstracts screening. After full-text reading, 34 articles were further excluded because of inappropriate outcomes, leaving 29 articles for data extraction. Four additional articles were retrieved for full text review after screening previous meta-analyses. As a result, 33 articles comprising 35 independent studies were initially retrieved, with two gender-stratified analyses by Lee et al. and Onitilo et al. identified as distinct datasets (Lee et al., 2011; Onitilo et al., 2014). The update searches were conducted in 20 March 2024 and resulted in the identification of 2 additional articles. Ultimately, 35 articles including 37 studies with 1,416,085 participants were integrated into this systematic review and meta-analysis comprising a total of 26 cohort studies, 5 case-control studies, and 6 nested case-control studies (Lee et al., 2011; Rosato et al., 2016; Seo et al., 2022; Tseng, 2017; Libby et al., 2009; Tsilidis et al., 2014; Onitilo et al., 2014; Bodmer et al., 2012; Bradley et al., 2018; Cardel et al., 2014; Chang et al., 2018; de Jong et al., 2017; Farmer et al., 2019; Krigel et al., 2022; Lee et al., 2021; Lin et al., 2015; Lopez et al., 2022; Murff et al., 2018; Rennert et al., 2020; Sehdev et al., 2015; Shin et al., 2020; Tseng, 2012; You et al., 2020; Zhang et al., 2022; Wang et al., 2013; Smiechowski et al., 2013; Yang et al., 2004; Oliveria et al., 2008; Ruiter et al., 2012; Oh et al., 2023; Chalhoub et al., 2023; Chung et al., 2008; Kanadiya et al., 2013; Cho et al., 2014; Kim et al., 2015). The search strategy and process for study selection are illustrated in Figure 1. Among these 37 studies, three only report effect sizes by doses of metformin instead of whether or not accepting metformin therapy, so these three studies were only analyzed in the dose-response meta-analysis (Bodmer et al., 2012; Chang et al., 2018; Tseng, 2012). The key characteristics of these studies are summarized in Table 1 and detailed characteristics for each study are available in Supplementary Table S3.

**TABLE 1:** Summary of key characteristics of included article.

First author	Country	Study type	Year	Total no. Of participants (n)	Risk expression	Adjusted effect size (95% CI)
Bodmer et al. (2012)	UK	nested case-control	2012	6439	OR	NA
Bradley et al. (2018)	USA	cohort	2018	47,351	HR	0.90 (0.76–1.07)
Cardel et al. (2014)	Denmark	nested case-control	2014	11,148	OR	0.83 (0.68–1.00)
Chang et al. (2018)	Taiwan	cohort	2018	47,597	HR	NA
de Jong et al. (2017)	Netherlands	cohort	2017	57,114	HR	0.89 (0.71–1.10)
Farmer et al. (2019)	UK	cohort	2019	55,629	HR	0.71 (0.43–1.18)
Krigel et al. (2022)	USA	cohort	2022	1869	OR	0.68 (0.51–0.92)
Lee et al. (2021)	Korea	cohort	2021	175,495	HR	0.66 (0.51–0.85)
				147,935		0.59 (0.37–0.92)
Lee et al. (2011)	Taiwan	cohort	2011	15,717	HR	0.36 (0.13–0.98)
Lin et al. (2015)	Taiwan	cohort	2015	34,823	HR	0.74 (0.53–1.03)
Lopez et al. (2022)	USA	cohort	2022	143,035	OR	0.79 (0.75–0.84)
Murff et al. (2018)	USA	cohort	2018	84,434	HR	0.89 (0.71–1.12)
Onitilo et al. (2014)	USA	cohort	2014	4956	HR	0.72 (0.37–1.38)
				4530		1.24 (0.62–2.47)
Rennert et al. (2020)	Israel	case-control	2020	8363	OR	0.754 (0.623–0.912)
Rosato et al. (2016)	Italy and Spain	case-control	2016	244	OR	0.47 (0.24–0.92)
Sehdev et al. (2015)	USA	case-control	2015	8046	OR	0.88 (0.77–1.00)
Seo et al. (2022)	Korea	cohort	2022	16,402	HR	0.58 (0.47–0.71)
Shin et al. (2020)	Korea	nested case-control	2020	8456	OR	0.96 (0.87–1.06)
Tseng (2017)	Taiwan	cohort	2017	32,662	HR	0.62 (0.53–0.74)
Tseng (2012)	Taiwan	cohort	2012	87,991	RR	NA
You et al. (2020)	Korea	cohort	2020	263,754	HR	0.865 (0.822–0.910)
Zhang et al. (2022)	Korea	cohort	2022	41,533	HR	0.88 (0.68–1.13)
Wang et al. (2013)	Taiwan	nested case-control	2013	10,767	OR	0.94 (0.73–1.21)
Smiechowski et al. (2013)	UK	nested case-control	2013	6444	RR	0.93 (0.73–1.18)
Yang et al. (2004)	UK	nested case-control	2004	1320	OR	1.0 (0.6–1.7)
Oliveria et al. (2008)	USA	cohort	2008	191,223	RR	0.67 (0.52–0.85)
Libby et al. (2009)	UK	cohort	2009	8170	HR	0.60 (0.38–0.94)
Ruiter et al. (2012)	Netherlands	cohort	2012	85,289	HR	0.91 (0.88–0.94)
Tsilidis et al. (2014)	UK	cohort	2014	69,748	HR	0.92 (0.76–1.13)
Oh et al. (2023)	Korea	cohort	2021	35,189	HR	0.481 (0.207–1.118)
Chalhoub et al. (2023)	Lebanon	case-control	2023	367	OR	0.363 (0.199–0.662)
Chung et al. (2008)	Korea	case-control	2008	200	OR	0.7 (0.3–1.4)
Kanadiya et al. (2013)	USA	cohort	2013	405	OR	0.55 (0.34–0.87)
Cho et al. (2014)	Korea	cohort	2014	3105	OR	0.73 (0.554–0.983)
Kim et al. (2015)	Korea	cohort	2015	240	RR	0.866 (0.453–1.623)

### Quality assessment

The average score of the NOS was 7.4 (ranging from 5 to 9). The methodological quality of eligible observational studies was moderate to high. Detailed scoring sheets are available in Supplementary Tables S4, S5.

### Overall meta-analysis

34 studies were included in overall meta-analysis. Considering the inherent time-to-event information encapsulated in HRs, the meta-analysis of HR and OR with 95% CI was separately conducted.

**FIGURE 2:**
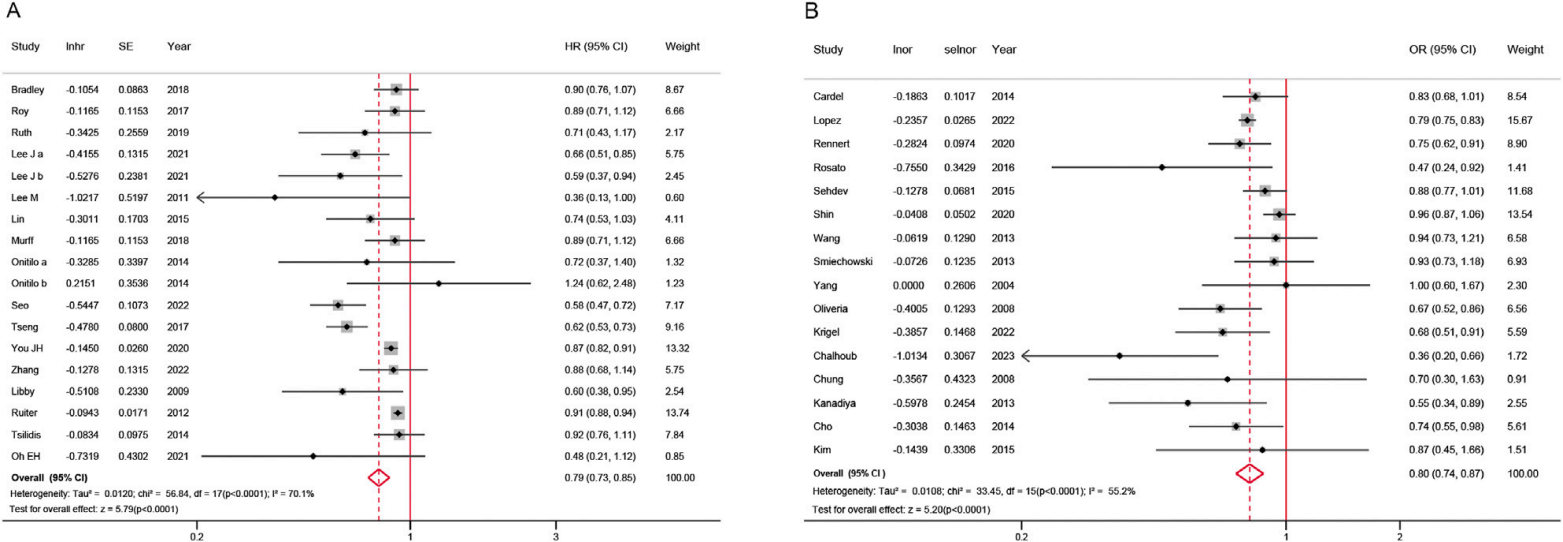
Forest plot of colorectal neoplasm risk among metformin users versus non metformin users. **(A)** Hazard ratios; **(B)** Odds ratios; CI: confidence interval; SE: standard error.

The overall meta-analysis was shown in Figure 2. A lower risk of colorectal neoplasm in metformin users compared to non-users was observed with the pooled HR of 0.79 (95% CI, 0.73–0.85) under the random-effects model (*I* ^*2*^ = 70.1%, *P* for heterogeneity <0.0001, 18 studies) Using the same model, the pooled OR obtained similar results with the value of 0.80 (95% CI, 0.74–0.87) and showed relatively high heterogeneity (*I* ^*2*^ = 55.2%, *P* for heterogeneity <0.0001, 16 studies). Both analyses showed statistical significance.

### Subgroup analysis

**FIGURE 3:**
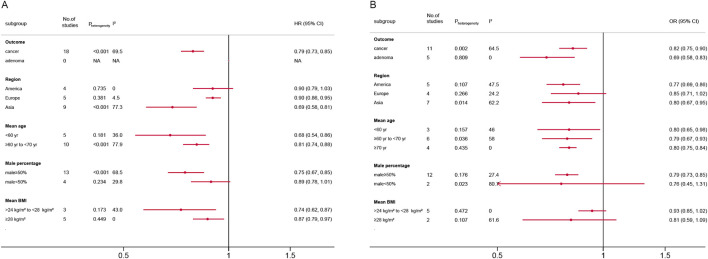
Forest plot of subgroup analysis according to different standards. **(A)** Hazard ratios; **(B)** Odds ratios; CI: confidence interval; BMI: body mass index.

Figure 3 present the subgroup analysis results. For HR analyses, metformin use was consistently associated with reduced colorectal neoplasm risk across most subgroups. Significant protective effects were observed in Asian populations (HR = 0.69, 95% CI, 0.58–0.81), younger than 60 (HR = 0.68, 95% CI, 0.54–0.86), male-predominant cohorts (HR = 0.75, 95% CI, 0.67–0.85) and the >24 to <28 kg/m^2^ BMI subgroup (HR = 0.74, 95% CI, 0.62–0.87). No significant associations were found in American cohorts (HR = 0.90, 95% CI, 0.79–1.03) or female-predominant groups (HR = 0.89, 95% CI, 0.78–1.01).

For OR analyses, metformin demonstrated a significant protective effect against both colorectal cancer (OR = 0.82, 95% CI, 0.75–0.90) and adenoma (OR = 0.69, 95% CI, 0.58–0.83). Protective effects were pronounced in Asian (OR = 0.80, 95% CI, 0.67–0.95) and American populations (OR = 0.77, 95% CI, 0.69–0.86), but non-significant in Europe (OR = 0.85, 95% CI, 0.71–1.02). All age subgroups showed similar risk reductions. Male-predominant cohorts exhibited robust effects (OR = 0.79, 95% CI, 0.73–0.85), whereas male <50% subgroups had inconclusive results (OR = 0.76, 95% CI, 0.45–1.31). No significant associations emerged in BMI subgroups. Detailed subgroup analyses plots were shown in Supplementary Figures S1–S10.

### Publication bias and sensitivity analyses

**FIGURE 4:**
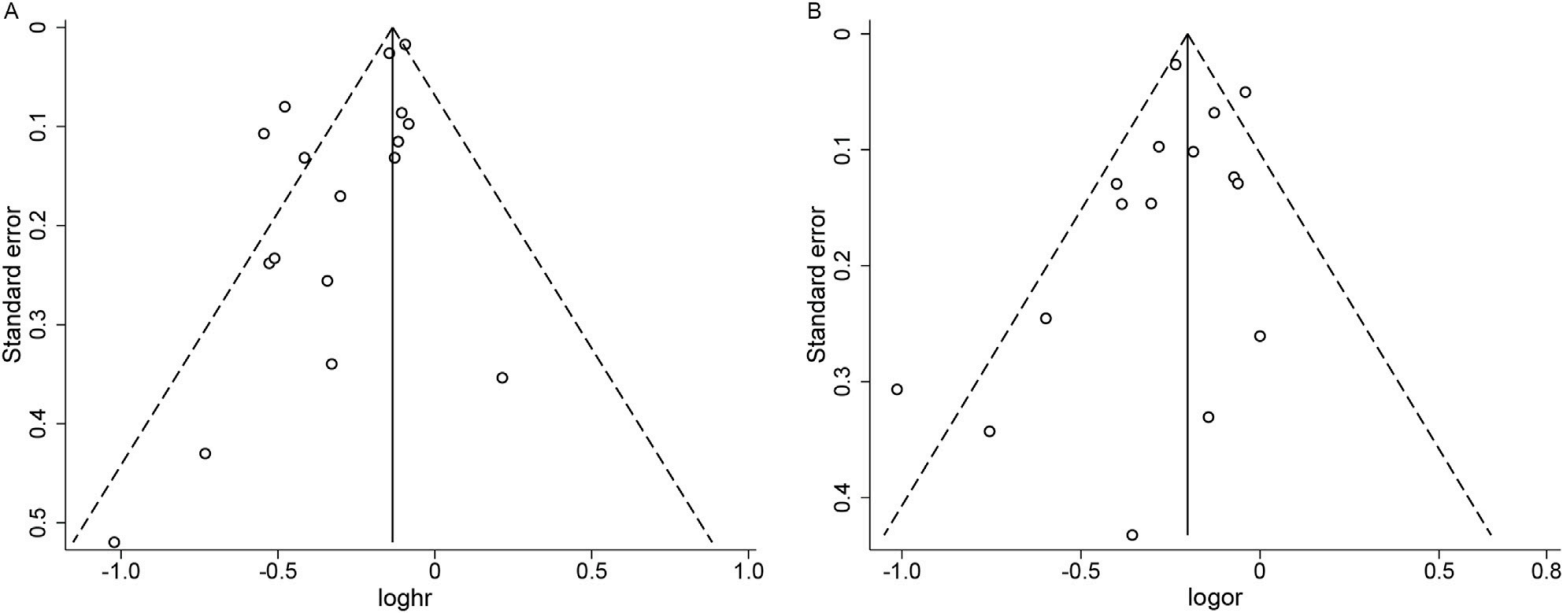
Funnel plot of published articles. **(A)** Hazard ratios; **(B)** Odds ratios.

In the analysis of publication bias for studies reporting HR, the funnel plot showed asymmetry, suggesting potential publication bias, which was corroborated by Egger’s test (*P* = 0.009) (Figure 4A). The trim-and-fill method revealed no evidence of missing studies (Supplementary Figure S11), lending support to the robustness of our findings; however, given the limited number of studies, the possibility of publication bias cannot be entirely excluded. For studies reporting OR, the funnel plot appeared approximately symmetric, with Egger’s test showing no evidence of publication bias (*P* = 0.312) (Figure 4B).

In sensitivity analyses, the results consistently demonstrated statistically significant inverse associations between metformin use and colorectal neoplasia risk (Table 2; Supplementary Figures S12–S17). Notably, all stratified estimates retained strong statistical significance, with HR ranging from 0.74 (95% CI, 0.65–0.83) to 0.79 (95% CI, 0.73–0.86) and OR spanning 0.78 (95% CI, 0.74–0.82) to 0.88 (95% CI, 0.83–0.94), indicating methodologically stable associations. In prespecified sensitivity analyses, all HRs originated from cohort designs and ORs uniformly employed other antidiabetic comparators, prespecified sensitivity analyses for these parameters were methodologically redundant and therefore excluded.

**TABLE 2:** Sensitivity analyses of pooled effect estimates.

Analysis criteria	Number of studies	Effect size (95% CI)	Heterogeneity (I^2^)	P-value
Hazard ratio				
Main Analysis	18	0.79 (0.73–0.85)	70.1	<0.0001
Other Antidiabetic Comparators^a^	14	0.74 (0.65–0.83)	68.4	<0.0001
High-Quality Studies (NOS ≥7)^b^	16	0.76 (0.68–0.84)	65.2	<0.0001
Diabetes-Specific Population^c^	17	0.79 (0.73–0.86)	70.9	<0.0001
Odds ratio				
Main Analysis	16	0.80 (0.74–0.87)	55.2	<0.0001
Cohort Studies Only^d^	6	0.78 (0.74–0.82)	0	<0.0001
High-Quality Studies (NOS ≥7)^b^	12	0.82 (0.79–0.86)	47.8	<0.0001
Diabetes-Specific Population^c^	12	0.88 (0.83–0.94)	36.1	<0.0001

^a^ Excluded studies with sulfonylurea monotherapy or no medication as comparators.

^b^ Excluded studies with Newcastle-Ottawa Scale (NOS) score <7.

^c^ Restricted to population with confirmed diabetes diagnosis.

^d^ Excluded non-cohort designs.

### Dose-response and duration–response analysis

**FIGURE 5:**
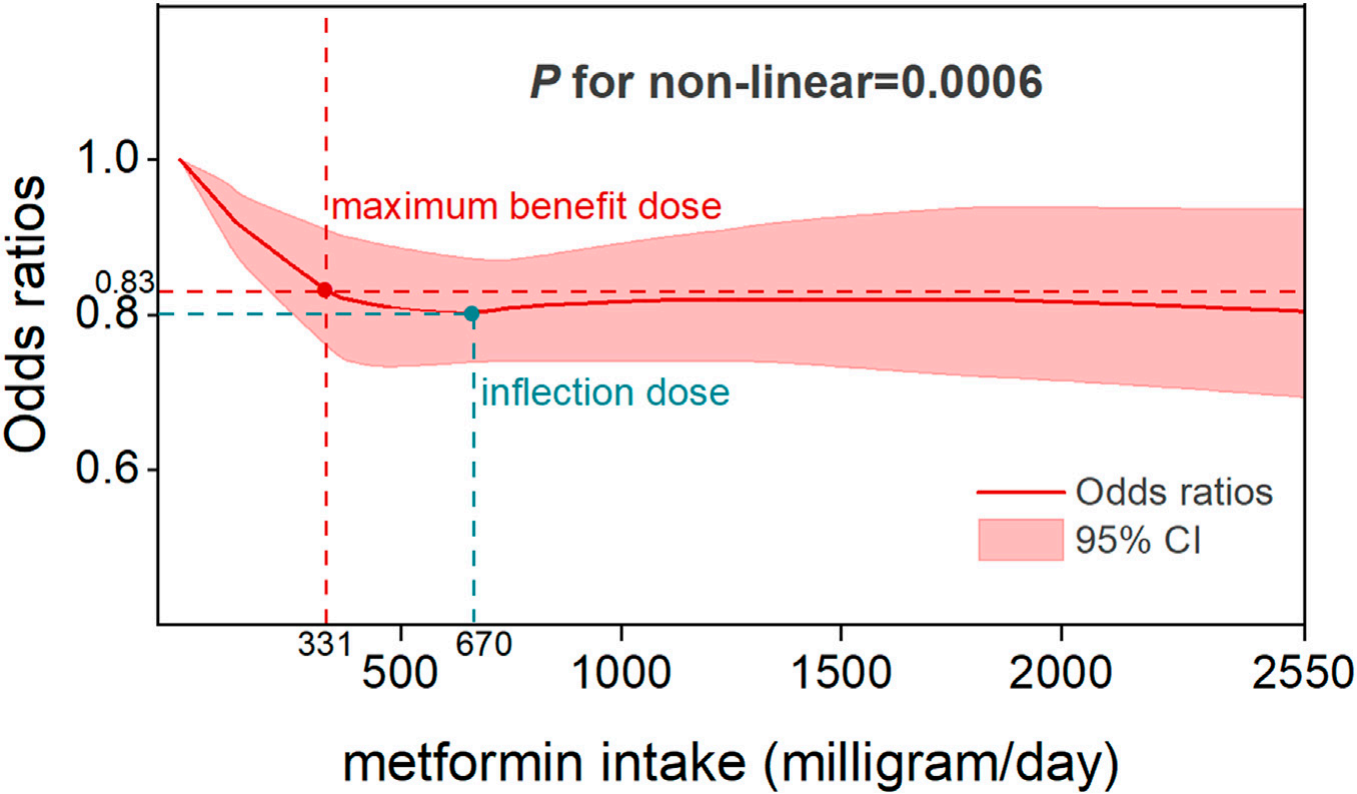
Dose-response relationship between metformin therapy and CRC risk.

**FIGURE 6:**
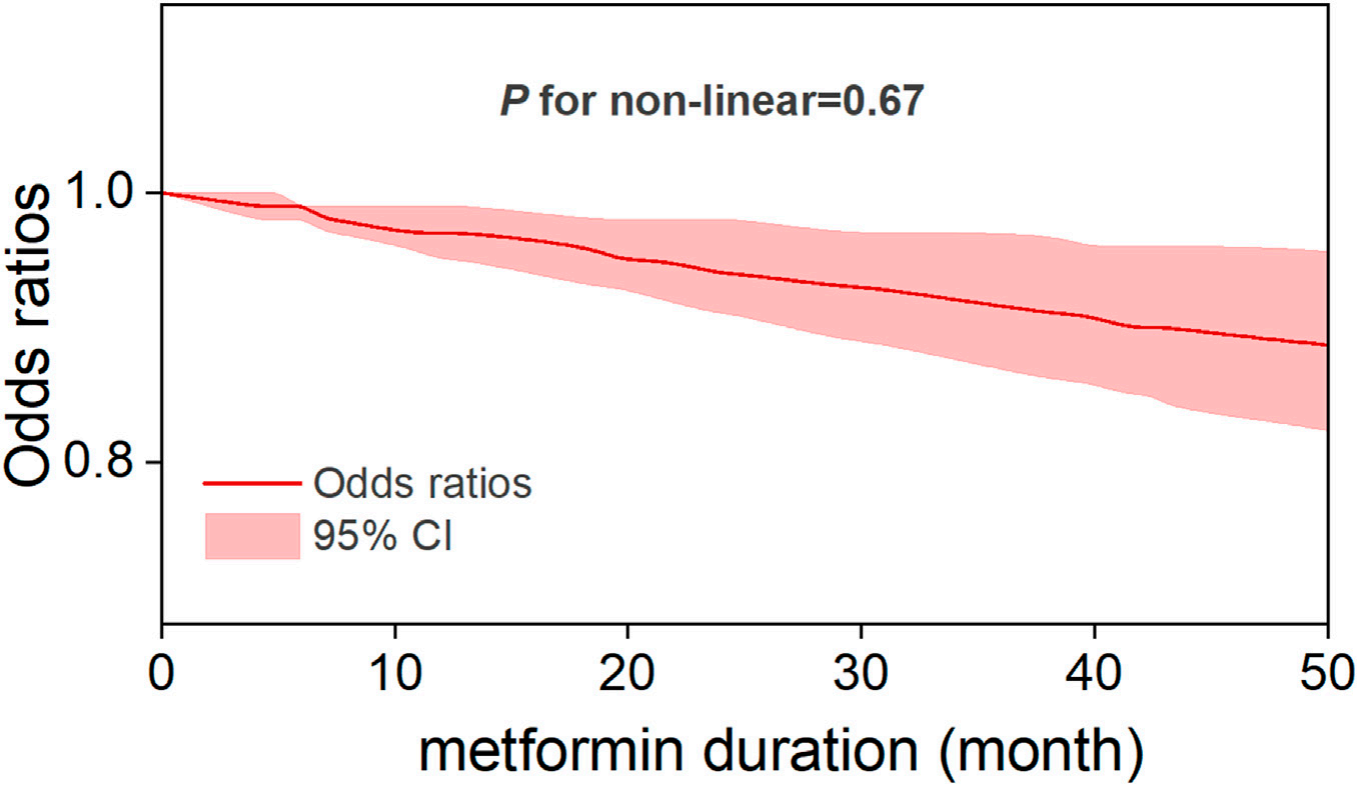
Duration-response relationship between metformin therapy and CRC risk.

Five studies reported OR estimates for intensity of metformin use and eleven for metformin duration. Detailed dose-response effect data of these studies were listed in Supplementary Table S6. The dose-response analysis revealed that there was an overall nonlinear relationship among metformin intake and the risk of CRC (*P* for non-linearity = 0.0006) (Figure 5). Specifically, the OR exhibited its steepest decline (OR = 0.83, 95% CI, 0.76–0.91) across the 0–331 mg/day dose range, defining the dose of maximum benefit. Beyond this threshold, the OR plateaued at its nadir (0.8, 95% CI, 0.74–0.87) until 670 mg/day, marking a critical inflection point beyond which the protective effect gradually diminished with increasing doses. A sensitivity analysis excluding studies with extreme doses showed that the non-linear association and the estimated optimal dose remained largely unchanged (Supplementary Figure S18). In the duration-response analysis, a linear relationship was observed between metformin treatment duration and colorectal cancer risk (*P* for non-linearity = 0.67), with study inclusion restricted to treatment durations ≤60 months. Specifically, each incremental year of metformin use was associated with a 3% reduction in CRC risk (OR = 0.97, 95% CI, 0.95–0.99), reflecting a modest yet statistically significant protective effect (Figure 6).

## Discussion

This study offers a comprehensive, up-to-date meta-analysis of the existing literature. The results stand for the potential chemoprevention of metformin and support the launch of metformin clinical trials for the chemoprevention of colorectal cancer.

In this systematic review and meta-analysis of moderate to high quality matched studies, we determined that metformin therapy was associated with a significant decrease in colorectal neoplasm risk. Dose-response and duration-response analyses demonstrated sustained protective effects of low-dose, long-term metformin therapy, which is consistent with the prophylactic dosage results reported by Higurashi et al. in their randomized phase III trial (Higurashi et al., 2016). Specifically, a nonlinear relation was observed between metformin intake and colorectal cancer risk, with the optimal protective effect observed at a low-dose threshold of 331 mg per day. This dose should not be viewed as a clinical recommendation, as standard regimens begin at ≥500 mg/day. While higher doses also showed risk reduction, the difference from 331 mg/day was not significant and may increase gastrointestinal side effects. This threshold therefore suggests that low doses can provide preventive benefits, whereas higher doses offer limited additional gain. Linear duration-response association revealed a 3% reduction in CRC risk per additional year of metformin use. To the best of our awareness, this is the first dose-response and duration-response meta-analysis that provide a comprehensive assessment to the relationship between the metformin therapy and primary prevention of CRC.

Previous evidence supported the antineoplastic properties of metformin on colorectal neoplasms. Zakikhani et al. first reported that metformin suppress the proliferation of HT-29 cells in a concentration-dependent manner (Zakikhani et al., 2008). Animal studies manifested that metformin decreased rectal aberrant crypt foci (ACF), adenomas and polyp generation in mouse model (Hosono et al., 2010a; Tomimoto et al., 2008). Further confirmation of a short-term randomized study demonstrated that metformin reduced the formation of CRC precancerous lesions by 40% (Hosono et al., 2010b). Similar to previous findings, a randomized phase III trial in post-polypectomy patients revealed that metformin in low dosage was efficacious in the prevention of colorectal adenomas (Higurashi et al., 2016). These findings are encouraging as metformin is frequently prescribed, safe, and affordable.

Metformin appears to exert a protective effect across multiple malignancies, though the strength of the association varies. Rangraze et al. reported that metformin use is associated with a reduced incidence of multiple cancers, including breast, lung, liver, and colorectal cancers, as well as overall cancer risk, with the most pronounced effects observed for breast and colorectal cancers (Rangraze et al., 2025). Laffusa et al. found that metformin use is associated with a markedly lower risk of cholangiocarcinoma, reducing incidence by about 62%–66% (Laffusa et al., 2023). In addition, metformin use was significantly associated with longer progression-free survival in patients with advanced pancreatic neuroendocrine tumors (Pusceddu et al., 2018). Compared with other malignancies, the protective effect of metformin on colorectal neoplasms appears to be comparable or slightly stronger and may reflect both systemic effects, such as improved insulin sensitivity and reduced inflammation, and tissue-specific mechanisms in the colorectal epithelium.

The current study exists some limitations. Firstly, all included studies were observational, which inherently carry risks of residual confounding. This may stem from the predominant focus of existing RCTs on metformin’s role in secondary prevention (particularly in preventing adenoma recurrence), which could be attributed to the practical challenges associated with extended follow-up periods in primary prevention studies. However, the exclusion of studies involving patients with prior adenoma resection was intentional, as such populations are subject to intensified surveillance and potential biases related to post-polypectomy inflammatory responses, which may confound the association between metformin and *de novo* tumor development. To mitigate potential biases, we calculated NOS for all studies and the scores revealed moderate to high quality. Future RCTs should address the challenges of long-term follow-up and prioritize investigating metformin’s role in primary prevention. Second, most included populations were generally defined by the absence of a prior diagnosis of colorectal cancer or adenomas rather than endoscopically confirmation. Thus, while we excluded recurrence studies in patients with a known history of adenomas, it remains possible that a proportion of participants had undetected adenomas at baseline. Nevertheless, our findings primarily reflect the preventive effect of metformin in populations without a documented adenoma history, which is highly relevant for primary prevention in real-world clinical settings. Third, despite subgroup and sensitivity analyses, substantial heterogeneity (I^2^ > 50%) remained, likely due to residual confounding from factors such as diabetes duration, glycemic control, adherence to metformin, lifestyle factors, concomitant medication use, and colorectal cancer screening behaviors, which were not consistently reported or harmonized across studies. These unmeasured factors may have contributed to the observed heterogeneity and could bias the pooled estimates. Moreover, our analysis was based on aggregate data extracted from published studies rather than individual participant data (IPD). The absence of IPD limited our ability to perform more refined stratified analyses according to important covariates such as age, sex, diabetes duration, and treatment adherence. Future studies should adopt more rigorous designs, including new-user active-comparator cohorts, individual participant data meta-analyses, and pragmatic randomized trials, to minimize bias and better clarify the causal role of metformin. Fourth, although the trim-and-fill analysis did not impute any missing studies in publication bias, the possibility of publication bias cannot be definitively excluded. Both funnel plots and Egger’s test have limited reliability when the number of included studies is fewer than 10–20, and their results may be unstable or inconclusive. Funnel plot asymmetry may also arise from other sources such as heterogeneity or chance rather than true publication bias. Therefore, while our trim-and-fill results suggest that the pooled HR estimates are relatively robust, we acknowledge that the assessment of publication bias remains methodologically limited. Lastly, the scarcity of standardized dose-response data precludes definitive conclusions about metformin’s chemopreventive thresholds. This knowledge gap hinders the establishment of optimal dosing strategies for cancer prevention. Extensive research is required to better understand the dose-response relationship.

## Conclusion

This meta-analysis provides evidence that metformin may serve as a potential primary chemopreventive agent for colorectal neoplasms. Additionally, our findings contribute to the understanding of the dose-response relationship between metformin use and the risk of colorectal cancer. To establish causality, future research should prioritize randomized controlled trials targeting primary prevention cohorts, with standardized protocols for metformin dosage, treatment duration, and outcome assessment.

## Data Availability

The original contributions presented in the study are included in the article/Supplementary Material, further inquiries can be directed to the corresponding author.
