# Supplementary material for: Metformin for primary prevention of colorectal neoplasms in adenoma-free populations: a systematic review and dose-response meta-analysis
Source: Front Pharmacol. 2025 Nov 19;16:1645387. doi: 10.3389/fphar.2025.1645387 (PMC12672328; doi:10.3389/fphar.2025.1645387)

**Supplementary Table 1 PRISMA checklist for the systematic review**

| **Section and Topic** | **Item #** | **Checklist item** | **Location where item is reported** |
| --- | --- | --- | --- |
| **TITLE** | | |  |
| Title | 1 | Identify the report as a systematic review. | Title |
| **ABSTRACT** | | |  |
| Abstract | 2 | See the PRISMA 2020 for Abstracts checklist. | Abstract |
| **INTRODUCTION** | | |  |
| Rationale | 3 | Describe the rationale for the review in the context of existing knowledge. | Intro 1–3 paras |
| Objectives | 4 | Provide an explicit statement of the objective(s) or question(s) the review addresses. | Intro 4th para |
| **METHODS** | | |  |
| Eligibility criteria | 5 | Specify the inclusion and exclusion criteria for the review and how studies were grouped for the syntheses. | Inclusion & exclusion criteria |
| Information sources | 6 | Specify all databases, registers, websites, organisations, reference lists and other sources searched or consulted to identify studies. Specify the date when each source was last searched or consulted. | Data sources & searches |
| Search strategy | 7 | Present the full search strategies for all databases, registers and websites, including any filters and limits used. | Supplementary Table 2 |
| Selection process | 8 | Specify the methods used to decide whether a study met the inclusion criteria of the review, including how many reviewers screened each record and each report retrieved, whether they worked independently, and if applicable, details of automation tools used in the process. | Data sources & searches |
| Data collection process | 9 | Specify the methods used to collect data from reports, including how many reviewers collected data from each report, whether they worked independently, any processes for obtaining or confirming data from study investigators, and if applicable, details of automation tools used in the process. | Data sources & searches |
| Data items | 10a | List and define all outcomes for which data were sought. Specify whether all results that were compatible with each outcome domain in each study were sought (e.g. for all measures, time points, analyses), and if not, the methods used to decide which results to collect. | Data extraction & quality assess 1st para |
|  | 10b | List and define all other variables for which data were sought (e.g. participant and intervention characteristics, funding sources). Describe any assumptions made about any missing or unclear information. | Data extraction & quality assess 1st para |
| Study risk of bias assessment | 11 | Specify the methods used to assess risk of bias in the included studies, including details of the tool(s) used, how many reviewers assessed each study and whether they worked independently, and if applicable, details of automation tools used in the process. | Data extraction & quality assess 2nd para |
| Effect measures | 12 | Specify for each outcome the effect measure(s) (e.g. risk ratio, mean difference) used in the synthesis or presentation of results. | Statistical methods |
| Synthesis methods | 13a | Describe the processes used to decide which studies were eligible for each synthesis (e.g. tabulating the study intervention characteristics and comparing against the planned groups for each synthesis (item #5)). | Table 1 |
|  | 13b | Describe any methods required to prepare the data for presentation or synthesis, such as handling of missing summary statistics, or data conversions. | Statistical methods |
|  | 13c | Describe any methods used to tabulate or visually display results of individual studies and syntheses. | Statistical methods |
|  | 13d | Describe any methods used to synthesize results and provide a rationale for the choice(s). If meta-analysis was performed, describe the model(s), method(s) to identify the presence and extent of statistical heterogeneity, and software package(s) used. | Statistical methods |
|  | 13e | Describe any methods used to explore possible causes of heterogeneity among study results (e.g. subgroup analysis, meta-regression). | Statistical methods |
|  | 13f | Describe any sensitivity analyses conducted to assess robustness of the synthesized results. | Statistical methods |
| Reporting bias assessment | 14 | Describe any methods used to assess risk of bias due to missing results in a synthesis (arising from reporting biases). | Statistical methods |
| Certainty assessment | 15 | Describe any methods used to assess certainty (or confidence) in the body of evidence for an outcome. | Statistical methods |
| **RESULTS** | | |  |
| Study selection | 16a | Describe the results of the search and selection process, from the number of records identified in the search to the number of studies included in the review, ideally using a flow diagram. | Figure 1 |
|  | 16b | Cite studies that might appear to meet the inclusion criteria, but which were excluded, and explain why they were excluded. | Figure 1 |
| Study characteristics | 17 | Cite each included study and present its characteristics. | Table 1 |
| Risk of bias in studies | 18 | Present assessments of risk of bias for each included study. | Figure 4 |
| Results of individual studies | 19 | For all outcomes, present, for each study: (a) summary statistics for each group (where appropriate) and (b) an effect estimate and its precision (e.g. confidence/credible interval), ideally using structured tables or plots. | Figure 2 |
| Results of syntheses | 20a | For each synthesis, briefly summarise the characteristics and risk of bias among contributing studies. | Overall meta-analysis |
|  | 20b | Present results of all statistical syntheses conducted. If meta-analysis was done, present for each the summary estimate and its precision (e.g. confidence/credible interval) and measures of statistical heterogeneity. If comparing groups, describe the direction of the effect. | Figure 3 |
|  | 20c | Present results of all investigations of possible causes of heterogeneity among study results. | Figure 3 |
|  | 20d | Present results of all sensitivity analyses conducted to assess the robustness of the synthesized results. | Table 2 |
| Reporting biases | 21 | Present assessments of risk of bias due to missing results (arising from reporting biases) for each synthesis assessed. | Figure 4 |
| Certainty of evidence | 22 | Present assessments of certainty (or confidence) in the body of evidence for each outcome assessed. | Supplementary Table 4-5 |
| **DISCUSSION** | | |  |
| Discussion | 23a | Provide a general interpretation of the results in the context of other evidence. | Discussion 1st para |
|  | 23b | Discuss any limitations of the evidence included in the review. | Discussion 5th para |
|  | 23c | Discuss any limitations of the review processes used. | Discussion 5th para |
|  | 23d | Discuss implications of the results for practice, policy, and future research. | Discussion 5th para |
| **OTHER INFORMATION** | | |  |
| Registration and protocol | 24a | Provide registration information for the review, including register name and registration number, or state that the review was not registered. | Methods 1st para |
|  | 24b | Indicate where the review protocol can be accessed, or state that a protocol was not prepared. | Not  performed |
|  | 24c | Describe and explain any amendments to information provided at registration or in the protocol. | Not  performed |
| Support | 25 | Describe sources of financial or non-financial support for the review, and the role of the funders or sponsors in the review. | Funding |
| Competing interests | 26 | Declare any competing interests of review authors. | Conflict of interest |
| Availability of data, code and other materials | 27 | Report which of the following are publicly available and where they can be found: template data collection forms; data extracted from included studies; data used for all analyses; analytic code; any other materials used in the review. | Data availability statement |

*From:*  Page MJ, McKenzie JE, Bossuyt PM, Boutron I, Hoffmann TC, Mulrow CD, et al. The PRISMA 2020 statement: an updated guideline for reporting systematic reviews. BMJ 2021;372:n71. doi: 10.1136/bmj.n71

**Supplementary Table 2 Detailed search strategy for** **the systematic review**

Search Strategy

| **Database** | **Search terms** | **Search results** |
| --- | --- | --- |
| Pubmed | ("Metformin"[MeSH Terms] OR "Dimethylbiguanidine"[Title/Abstract] OR "Dimethylguanylguanidine"[Title/Abstract] OR "Glucophage"[Title/Abstract] OR "metformin hydrochloride"[Title/Abstract] OR "metformin hcl"[Title/Abstract]) AND ("colorectal neoplasms"[MeSH Terms] OR "colonic neoplasms"[MeSH Terms] OR "rectal neoplasms"[MeSH Terms] OR (("Colon"[MeSH Terms] OR "administration, rectal"[MeSH Terms] OR "colonic"[Title/Abstract] OR "Colon"[Title/Abstract] OR "colon s"[Title/Abstract] OR "colons"[Title/Abstract] OR "colonal"[Title/Abstract] OR "colonically"[Title/Abstract] OR "colonitis"[Title/Abstract] OR "colorectal"[Title/Abstract] OR "rectal"[Title/Abstract] OR "rectal administration"[Title/Abstract] OR "administration rectal"[Title/Abstract])  AND  ("carcinoma"[MeSH Terms] OR "adenoma"[MeSH Terms] OR "neoplasm"[Title/Abstract] OR "Neoplasms"[Title/Abstract] OR "cancer"[Title/Abstract] OR "carcinoma"[Title/Abstract] OR "tumour"[Title/Abstract] OR "adenoma"[Title/Abstract] OR "adenomas"[Title/Abstract] OR "CRC"[Title/Abstract]))) | 11 November 2022, 308  20 March 2024, 34 updated |
| Embase | ('metformin'/exp OR dimethylbiguanidine:ab,ti OR dimethylguanylguanidine:ab,ti OR glucophage:ab,ti OR 'metformin hydrochloride':ab,ti OR 'metformin hcl':ab,ti)  AND  ('colorectal neoplasms'/exp OR 'colonic neoplasms'/exp OR 'rectal neoplasms'/exp OR (('colon'/exp OR 'administration, rectal'/exp OR colonic:ab,ti OR colon:ab,ti OR 'colon s':ab,ti OR colons:ab,ti OR colonal:ab,ti OR colonically:ab,ti OR colonitis:ab,ti OR colorectal:ab,ti OR rectal:ab,ti OR 'rectal administration':ab,ti OR 'administration rectal':ab,ti)  AND  ('carcinoma'/exp OR 'adenoma'/exp OR neoplasm:ab,ti OR neoplasms:ab,ti OR cancer:ab,ti OR carcinoma:ab,ti OR tumour:ab,ti OR adenoma:ab,ti OR adenomas:ab,ti))) | 11 November 2022, 1713  20 March 2024, 425 updated |

**Supplementary Table 3 Complete and Detailed Characteristics of Included Studies**

| First author | Country | Study type | Year | Total No. of participants  (n) | Male  (%) | Age  (y)  Mean±SD  /median (interquartile range) | BMI  (kg/m^2^)  mean±SD  /median (interquartile range) | Comparison | Risk expression | Adjusted effect size (95% CI) | Adjusted variables |
| --- | --- | --- | --- | --- | --- | --- | --- | --- | --- | --- | --- |
| Bodmer | UK | nested case-control | 2012 | 6439 | Cases:63.3  Controls:63.3 | Cases:70.2±8.6  Controls:70.2±8.6 | NA | Metformin vs non-metformin | OR | NA | Matching variables: calendar time, age, sex, general practice, number of years of active history prior to the index date  Adjusted variables: BMI, smoking, diabetes duration, prior use of aspirin, NSAID, and statins |
| Bradley | USA | cohort | 2018 | 47351 | Exposed:52.3  Reference:55.3 | 61.85 | Exposed:29.8 (26.3-34.2)  Reference:27.9 (24.8-31.9) | Metformin vs non-metformin | HR | 0.90 (0.76-1.07) | gender, race/ethnicity, birth year, diabetes duration, BMI, alcohol use, smoking status, CCI, education, income level, creatinine, HbA1c, history of lower endoscopy, first lower endoscopy after baseline use of other types of diabetes medications |
| Cardel | Denmark | nested case-control | 2014 | 11148 | Cases:60.7  Controls:53.6 | Cases:73 (66-80)  Controls:69 (61-77) | NA | Metformin vs non-metformin | OR | 0.83 (0.68-1.00) | age, gender, calendar year, tobacco, obesity, alcohol use, aspirin, NSAIDs, statins, glibenclamide, and gliclazide |
| Chang | Taiwan | cohort | 2018 | 47597 | 53.1 | 54.39±14.19 | NA | Metformin vs non-metformin | HR | NA | age, sex, and CCI score |
| Roy | Netherlands | cohort | 2017 | 57114 | Exposed:48.8  Reference:47.0 | Exposed:63.5±12.7  Reference:67.0±12.9 | NA | Metformin vs non-metformin | HR | 0.89 (0.71-1.10) | age, sex, use of insulin, history of hospitalization, duration of diabetes, and year of start of follow-up |
| Ruth | UK | cohort | 2019 | 55629 | 56.4 | 61.7±12 | 31.8±6.3 | Metformin vs no medication | HR | 0.71 (0.43-1.18) | age, gender, smoking status and alcohol status, year of onset of diabetes, HbA1c, BMI, use of other medications in previous year, history of chronic kidney disease and cardiovascular disease |
| Krigel | USA | cohort | 2022 | 1869 | 97.2 | 62.5±7.88 | 28.06 | Metformin vs non-metformin | OR | 0.68 (0.51-0.92) | age, race, ethnicity, BMI, tobacco smoking history, colonoscopy indication, exposure to other medications including aspirin, NSAIDs, statins, and insulin |
| Lee J | Korea | cohort | 2021 | 175495 | 100 | Exposed:55.0±7.7  Reference:52.4±8.0 | Exposed:24.6±2.8  Reference:24.1±3.1 | Metformin vs non-metformin | HR | 0.66 (0.51-0.85) | age, smoking status, drinking status, physical activity, BMI, systolic blood pressure, past hypertension history, total cholesterol, alanine aminotransferase, income status and glucose levels |
|  |  |  |  | 147935 | 0 | Exposed:58.0±7.3  Reference:55.8±8.3 | Exposed:25.2±3.3  Reference:24.5±3.3 |  |  | 0.59 (0.37-0.92) |  |
| Lee M | Taiwan | cohort | 2011 | 15717 | Exposed:54.9  Reference:54.6 | 55.9 | NA | Metformin vs non-metformin | HR | 0.36 (0.13-0.98) | age group, gender, other oral anti-hyperglycemic medication, CCI score, and duration of metformin exposure |
| Lin | Taiwan | cohort | 2015 | 34823 | NA | NA | NA | Metformin vs non-metformin | HR | 0.74 (0.53-1.03) | age, sex, hypertension, dyslipidemia, obesity, gout, hepatitis B, hepatitis C, liver cirrhosis, and duration of ADT exposure |
| Lopez | USA | cohort | 2022 | 143035 | 100 | 75 | NA | Metformin vs non-metformin | OR | 0.79 (0.75-0.84) | age, race, hypogonadism, hypertension, diabetes, use of insulin, muscular wasting, malaise and fatigue, osteoporosis, erectile dysfunction, depression, anterior pituitary disorder, education, percentage of adults below poverty line, patients' primary care, colorectal cancer screening, and mutual adjustment for TTh and metformin. |
| Murff | USA | cohort | 2018 | 84434 | Exposed:97.2  Reference:97.2 | Exposed:66.2 (57.6-74.7)  Reference:65.4 (57.3-74.6) | Exposed:30.7 (27.5-34.7)  Reference:30.7 (27.5-34.7) | Metformin vs sulfonylurea | HR | 0.89 (0.71-1.12) | age, sex, race, date of cohort entry, BMI, blood pressure, glomerular filtration rate, HbA1c, low-density lipoprotein levels, smoking status, select medications, co-morbid illnesses, number of medications, and number of outpatient visits |
| Onitilo | USA | cohort | 2014 | 4956 | 100 | 61.7 (52.3-71.1) | 33.7 (28.7-37.4) | Metformin vs non-metformin | HR | 0.72 (0.37-1.38) | age, BMI, DM diagnosis date, insurance status, comorbidities, smoking history, and location of residence |
|  |  |  |  | 4530 | 0 |  |  |  |  | 1.24 (0.62-2.47) |  |
| Rennert | Israel | case-control | 2020 | 8363 | Cases:52.2  Controls:52.4 | Cases:70.9±11.50  Controls:71.7±11.40 | NA | Metformin vs non-metformin | OR | 0.754 (0.623-0.912) | Matching variables: age, gender, ethnicity and residence  Adjusted variables: age, history of diabetes, use of other anti-diabetes drugs, physical activity, consumption of vegetables, fruits, use of aspirin and statins, first degree family history of colorectal cancer |
| Rosato | Italy and Spain | case-control | 2016 | 244 | NA | NA | NA | Metformin vs non-metformin | OR | 0.47 (0.24-0.92) | study center, sex, age, education, tobacco smoking, alcohol drinking, BMI, physical activity, statin use, and aspirin use |
| Sehdev | USA | case-control | 2015 | 8046 | Cases:59.77  Controls:59.77 | Cases:57.37±5.51  Controls:55.23±5.69 | NA | Metformin vs non-metformin | OR | 0.88 (0.77-1.00) | Matching variables: age, sex, and geographical region  Adjusted variables: obesity, inflammatory bowel disease, polycystic ovary disease, coronary artery disease, CCI, NSAIDs, sulfonylureas, thiazolidinediones, statins and insulin |
| Seo | Korea | cohort | 2022 | 16402 | Exposed:50.9  Reference:51.5 | 62.69 | NA | Metformin vs non-metformin | HR | 0.58 (0.47-0.71) | age group, sex, index year, CCI, all recorded drugs before 1 year of cohort entry, all recorded diagnostic before 1 year of cohort entry |
| Shin | Korea | nested case-control | 2020 | 8456 | Cases:65.30  Controls：23.46 | Cases:61.31±9.47  Controls:61.42±9.14 | Cases:24.74±3.25  Controls:24.70±3.25 | Metformin vs non-metformin | OR | 0.96 (0.87-1.06) | Matching variables: age, sex, the year of DM diagnosis, BMI, FPG level,  Adjusted variables: smoking, drinking, waist circumference, exercise, and insulin use, and the time from DM diagnosis to index date |
| Tseng | Taiwan | cohort | 2017 | 32662 | Exposed:57.2  Reference:57.4 | Exposed:63.7±9.9  Reference:63.6±10.4 | NA | Metformin vs non-metformin | HR | 0.62 (0.53-0.74) | age, gender, occupation and residential region, and factors that might be correlated with metformin use, diabetes severity and cancer risk, region of residence and occupation, major comorbidities, diabetes-related complications, antidiabetic drugs, diagnoses that may be associated with cancer risk, medications commonly used in diabetes patients that might potentially affect cancer risk |
| Tseng | Taiwan | cohort | 2012 | 87991 | NA | NA | NA | Metformin vs non-metformin | RR | NA | age, sex, diabetes status, dyslipidemia, obesity,  hypertension, chronic  obstructive, pulmonary  disease, asthma, stroke, nephronpathy, ischemic heart disease, peripheral arterial disease, eye disease, statins, fibrates, angiotensin-converting enzyme inhibitors and/or angiotensin receptor blockers, calcium channel blockers, aspirin, dipyridamole,clopidogrel/ticlopidine, nonsteroidal anti-inflammatory drugs, sulfonylurea, metformin, insulin, acarbose,thiazolidinedione,  region of residence, occupation, and potential colon cancer detection examinations |
| You | Korea | cohort | 2020 | 263754 | Exposed:49.9  Reference:50.9 | Exposed:60.7±15.4  Reference:60.9±13.9 | NA | Metformin vs non-metformin | HR | 0.865 (0.822-0.910) | age, sex, economic status, region of residency, and antidiabetic medications |
| Zhang | Korea | cohort | 2022 | 41533 | 73.3 | 58.0±10.1 | 25.3±3.1 | Metformin vs non-metformin | HR | 0.88 (0.68-1.13) | age, gender, smoking status, alcohol drinking status, exercise status, BMI, and fasting blood glucose |
| Wang | Taiwan | nested case-control | 2013 | 10767 | NA | NA | NA | Metformin vs non-metformin | OR | 0.94 (0.73-1.21) | age, sex, occupation |
| Smiechowski | UK | nested case-control | 2013 | 6444 | Cases:63.3  Controls:63.6 | Cases:72.8±8.7  Controls:72.5±8.5 | 28.29 | Metformin vs non-metformin | RR | 0.93 (0.73-1.18) | Matching variables: age, sex, calendar year of cohort entry and duration of follow-up  Adjusted variables: obesity, smoking, statins, nonsteroidal anti-inflammatory drugs, aspirin, excessive alcohol use, HbA1c, diabetes duration, cholecystectomy, inflammatory bowel diseases, referrals to colonoscopy, referrals to sigmoidoscopy, history of polyps, previous cancer, use of sulfonylureas, thiazolidinediones, insulins, and other antidiabetic agents |
| Yang | UK | nested case-control | 2004 | 1320 | Cases:64  Controls:49.4 | Cases:74.9±8.5  Controls:74.9±8.4 | 26.74 | Metformin vs non-metformin | OR | 1.0 (0.6-1.7) | Matching variables: year of birth, calendar period, duration of follow-up in the database prior to the case index date  Adjusted variables: sex, history of cholecystectomy, smoking, duration of type 2 diabetes mellitus, BMI, NSAID/aspirin use |
| Oliveria | USA | cohort | 2008 | 191223 | 51 | 56 | NA | Metformin vs non-metformin | RR | 0.67 (0.52-0.85) | age, gender, selected cancer risk factors  age, gender, and selected cancer risk factors: history of polyps, ulcerative colitis, Crohn’s disease |
| Libby | UK | cohort | 2009 | 8170 | Exposed:54.1  Reference:54.8 | 67.16 | Exposed:30.7±3.5  Reference:28.6±3.1 | Metformin vs non-metformin | HR | 0.60 (0.38-0.94) | age, sex, smoking, deprivation, BMI, A1C, insulin use, and sulfonylurea use |
| Ruiter | Netherlands | cohort | 2012 | 85289 | Exposed:46.4  Reference:48.2 | Exposed:61.8±13.4  Reference:65.6±13.8 | NA | Metformin vs sulfonylurea | HR | 0.91 (0.88-0.94) | age, sex, year in which the first OGLD prescription was dispensed, number of unique drugs used and number of hospitalizations in the year |
| Tsilidis | UK | cohort | 2014 | 69748 | Exposed:56.1  Reference:57.9 | 62.23 | 29.22 | Metformin vs sulfonylurea | HR | 0.92 (0.76-1.13) | age, sex, smoking status, BMI, alcohol consumption, use of aspirin or NSAIDs, statins, diabetes duration, and year of first antidiabetes prescription |
| Oh EH | Korea | cohort | 2021 | 35189 | Total:60.7 | 41（29-54） | NA | Metformin vs non-metformin | HR | 0.481 (0.207–1.118) | age, sex, comorbidity, and ulcerative colitis treatment group |
| Chalhoub | Lebanon | case-control | 2023 | 367 | 63.2 | NA | NA | Metformin vs non-metformin | OR | 0.363 (0.199–0.662) | comorbid factors and risk/protective factors for colorectal cancer |
| Chung | Korea | case-control | 2008 | 200 | Cases:52  Controls:52 | Cases:66.8±9.4  Controls:66.2±10.9 | Cases:24.5±3.7  Controls:23.9±3.7 | Metformin vs non-metformin | OR | 0.7 (0.3-1.4) | age, gender, BMI, duration of DM, serum levels of HbA1c and lipids, use of insulin and aspirin |
| Kanadiya | USA | cohort | 2013 | 405 | 49.6 | 63.96±9.19 | NA | Metformin vs sulfonylurea | OR | 0.55 (0.34-0.87) | age, gender, smoking status, use of alcohol |
| Cho | Korea | cohort | 2014 | 3105 | Exposed:60  Reference:56.4 | Exposed:60.1±10.8  Reference:63.9±12.0 | Exposed:25.0±3.7  Reference:24.3±3.6 | Metformin vs non-metformin | OR | 0.73 (0.554-0.983) | age, sex, BMI, triglyceride, glycated hemoglobin, aspirin use, statin use, smoking, diabetes mellitus duration |
| Kim | Korea | cohort | 2015 | 240 | Exposed:69.5  Reference:67.4 | Exposed:58.8±9.9  Reference:61.6±10.3 | Exposed:25.2±3.0  Reference:25.2±3.3 | Metformin vs non-metformin | RR | 0.866 (0.453-1.623) | age, gender, BMI, smoking status, use of aspirin and alcohol |

Abbreviations: SD, standard deviation; NA, not available; NSAIDs, non-steroidal anti-inflammatory drugs; CCI: Charlson comorbidity index; ADT, anti-diabetic therapy; TTH, the independent and joint association of metformin and testosterone replacement therapy; OGLD, oral glucose-lowering drug.

**Supplementary Table 4 The Newcastle-Ottawa Scale (NOS) assessing the quality of case-control studies**

| 1st author year | Year | Selection | | | | Comparability | Exposure | | | Total quality scores |
| --- | --- | --- | --- | --- | --- | --- | --- | --- | --- | --- |
|  |  | Is the case definition adequate | Representativeness of the cases | Selection of Controls | Definition of Controls | Comparability of cases and controls on the basis of the design or analysis^a^ | Ascertainment of exposure | Same method of ascertainment for cases and controls | Non-Response rate |  |
| Chalhoub | 2023 | ★ | - | ★ | ★ | ★ | ★ | ★ | - | 6 |
| Rennert | 2020 | ★ | ★ | ★ | ★ | ★★ | ★ | ★ | ★ | 9 |
| Rosato | 2016 | ★ | - | ★ | ★ | ★★ | - | - | ★ | 6 |
| Sehdev | 2015 | ★ | ★ | ★ | ★ | ★★ | ★ | ★ | ★ | 9 |
| Chung | 2008 | ★ | - | ★ | ★ | ★ | ★ | ★ | ★ | 7 |

^a^ Score one point if the study controls for the most important factor, score two points if the study controls for any additional factor

**Supplementary Table 5 The Newcastle-Ottawa Scale (NOS) assessing the quality of cohort and nested case-control studies**

| 1st author year | Year | Selection | | | | Comparability | Outcome | | | Total quality scores |
| --- | --- | --- | --- | --- | --- | --- | --- | --- | --- | --- |
|  |  | Representativeness of exposed cohort | Selection of non-exposed cohort | Ascertain merit of exposure | Demonstration that outcome of interest was not present at start of study | Comparability of cohorts on the basis of the design or analysis^a^ | Assessment of outcome | Follow-up long enough for outcomes to occur^b^ | Adequacy of follow up of cohorts^c^ |  |
| Krigel | 2022 | - | ★ | ★ | ★ | ★★ | ★ | - | ★ | 7 |
| Lopez | 2022 | - | ★ | ★ | ★ | ★★ | ★ | - | ★ | 7 |
| Seo | 2022 | ★ | ★ | ★ | ★ | ★★ | ★ | - | ★ | 8 |
| Zhang | 2022 | - | ★ | ★ | ★ | ★★ | ★ | ★ | ★ | 8 |
| Oh EH | 2021 | - | ★ | ★ | ★ | ★ | ★ | - | ★ | 6 |
| Lee J | 2021 | ★ | ★ | ★ | ★ | ★★ | ★ | ★ | ★ | 9 |
| Lee M | 2021 | ★ | ★ | ★ | ★ | ★★ | ★ | - | ★ | 8 |
| Shin | 2020 | ★ | ★ | ★ | ★ | ★★ | ★ | - | ★ | 8 |
| You JH | 2020 | ★ | ★ | ★ | ★ | ★★ | ★ | ★ | ★ | 9 |
| Ruth | 2019 | ★ | ★ | ★ | ★ | ★★ | ★ | - | - | 7 |
| Bradley | 2018 | - | ★ | ★ | ★ | ★★ | ★ | ★ | ★ | 8 |
| Chang | 2018 | ★ | ★ | ★ | ★ | ★ | ★ | ★ | ★ | 8 |
| Murff | 2018 | - | ★ | ★ | ★ | ★★ | ★ | - | ★ | 7 |
| Roy | 2017 | ★ | ★ | ★ | ★ | ★ | ★ | - | ★ | 7 |
| Tseng | 2017 | ★ | ★ | ★ | ★ | ★★ | ★ | ★ | ★ | 9 |
| Lin | 2015 | ★ | ★ | ★ | ★ | ★★ | ★ | - | - | 7 |
| Kim | 2015 | - | ★ | ★ | - | ★ | ★ | - | ★ | 5 |
| Cardel | 2014 | - | ★ | ★ | ★ | ★★ | ★ | - | ★ | 7 |
| Tsilidis | 2014 | - | ★ | ★ | ★ | ★★ | ★ | ★ | - | 7 |
| Onitilo | 2014 | - | ★ | ★ | ★ | ★★ | ★ | ★ | ★ | 8 |
| Cho | 2014 | - | ★ | ★ | ★ | ★★ | ★ | - | ★ | 7 |
| Wang | 2013 | ★ | ★ | ★ | ★ | ★ | ★ | - | ★ | 7 |
| Smiechowski | 2013 | - | ★ | ★ | ★ | ★★ | ★ | - | ★ | 7 |
| Kanadiya | 2013 | - | ★ | ★ | ★ | ★ | ★ | - | ★ | 6 |
| Bodmer | 2012 | ★ | ★ | ★ | ★ | ★★ | ★ | - | ★ | 8 |
| Tseng | 2012 | ★ | ★ | ★ | ★ | ★★ | ★ | - | ★ | 8 |
| Ruiter | 2012 | ★ | ★ | ★ | ★ | ★ | ★ | - | - | 6 |
| Libby | 2009 | ★ | ★ | ★ | ★ | ★★ | ★ | - | ★ | 8 |
| Oliveria | 2008 | ★ | ★ | ★ | ★ | ★★ | ★ | - | - | 7 |
| Yang | 2004 | ★ | ★ | ★ | ★ | ★★ | ★ | ★ | - | 8 |

^a^ Score one point if the study controls for the most important factor, score two points if the study controls for any additional factor

^b^ Score one point if the study follow-up is longer than 5 years

^c^ Score one point if the study has a 90% follow-up rate, or 70% follow-up rate and describes the loss to follow-up

**Supplementary Figure 1** **Forest plot of hazard ratios for subgroups stratified by outcome**

**
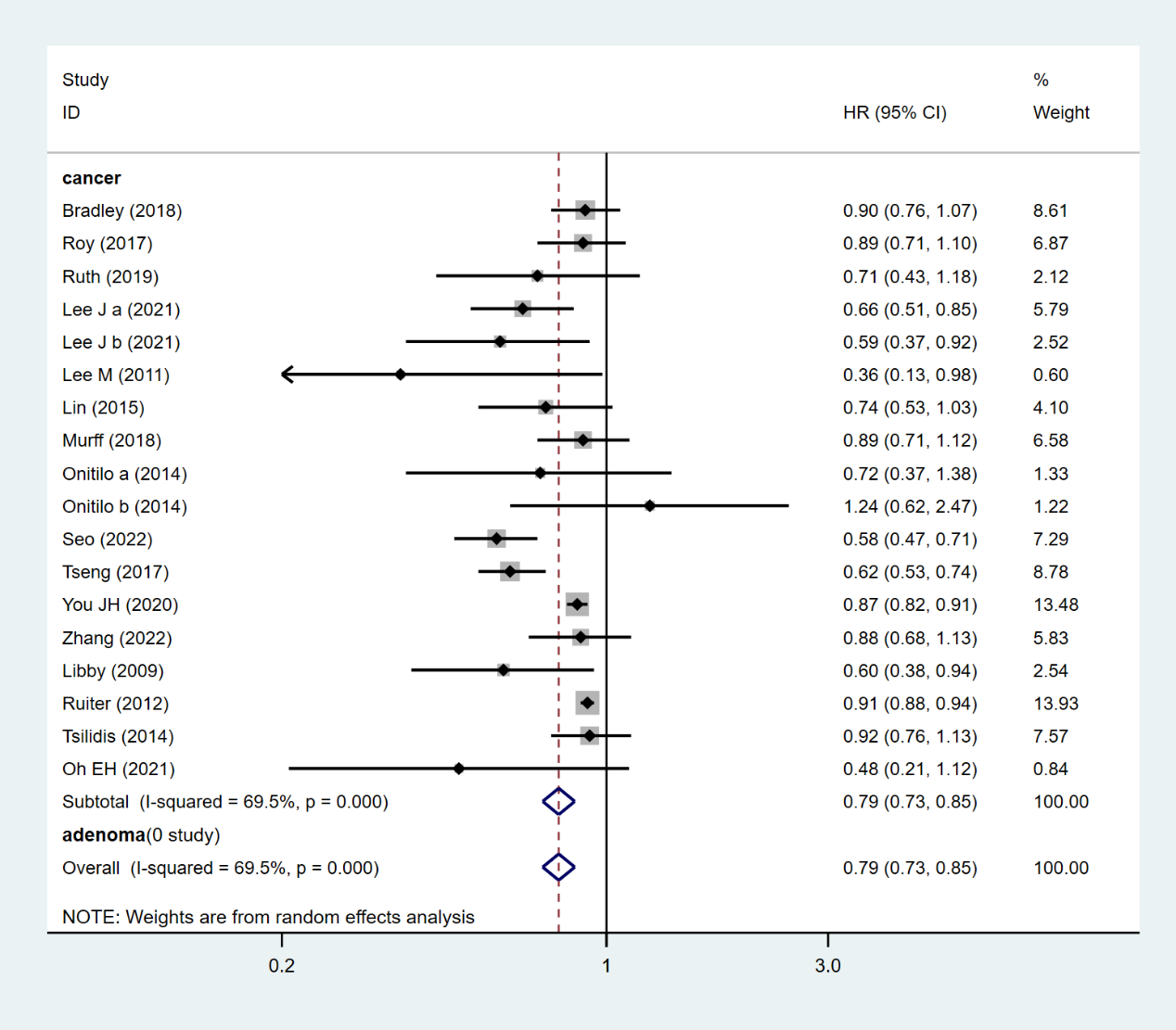
**

**Supplementary Figure 2 Forest plot of hazard ratios for subgroups stratified by region**

**
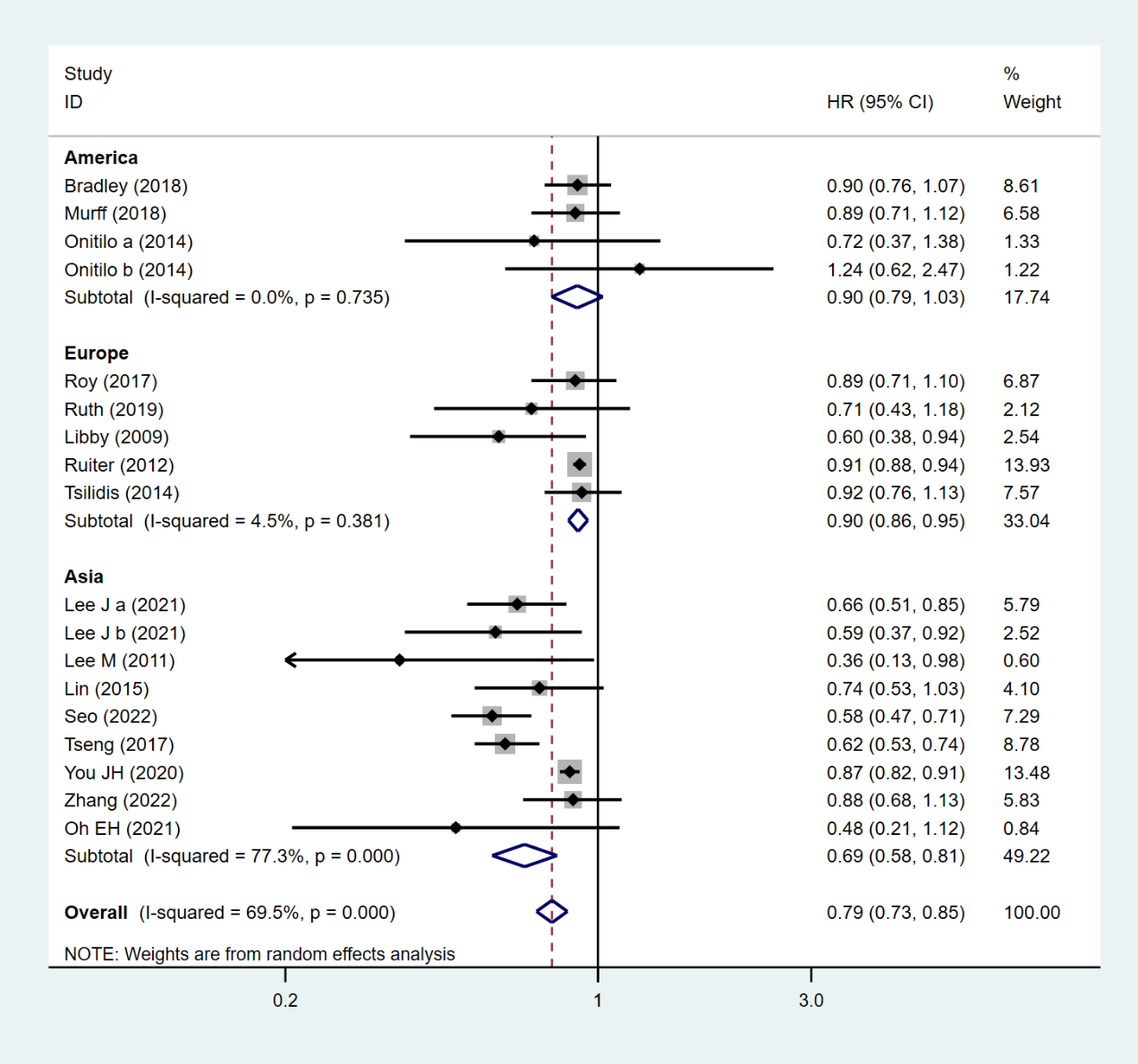
**

**Supplementary Figure 3 Forest plot of hazard ratios for subgroups stratified by mean age**

**
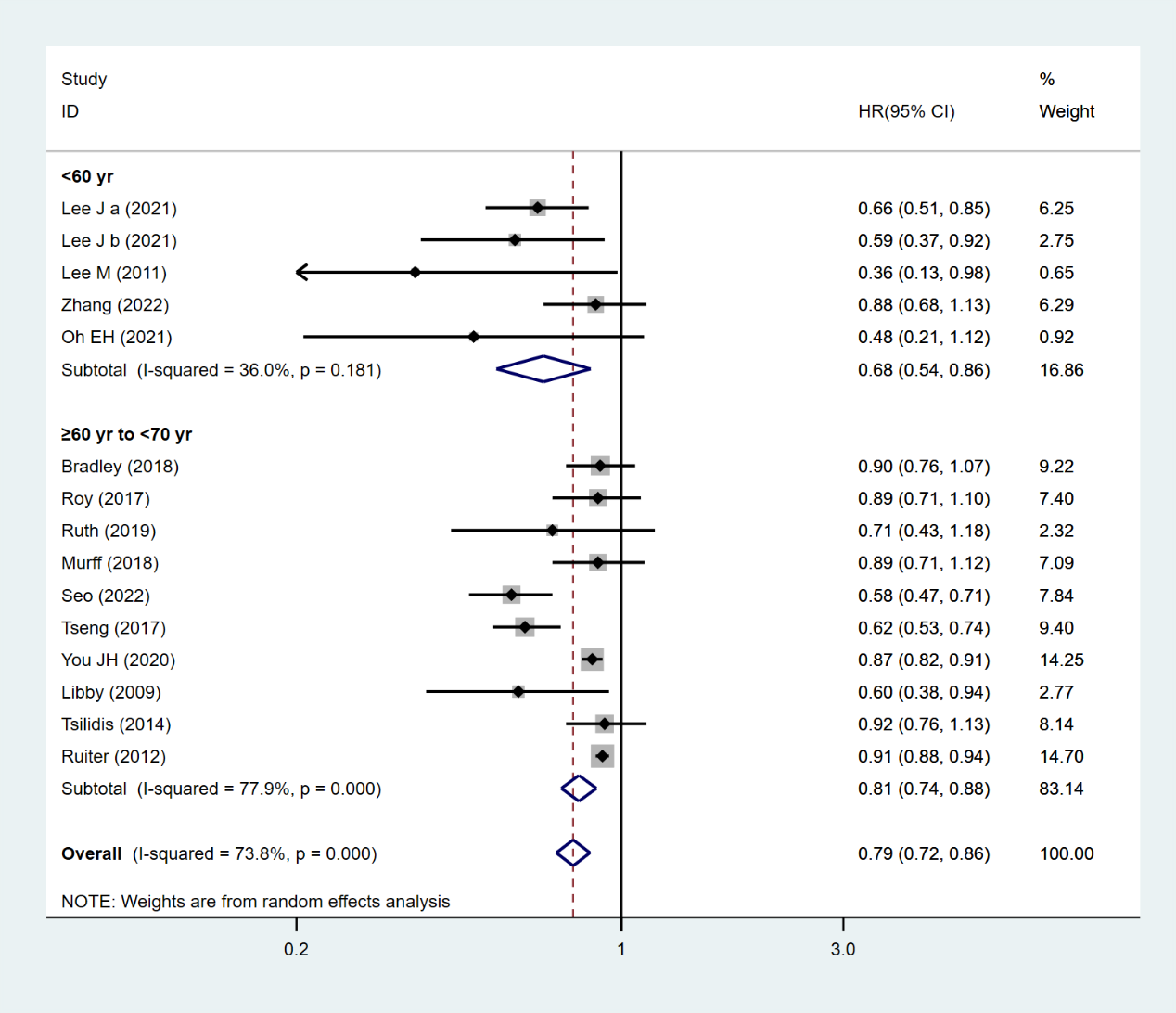
**

**Supplementary Figure 4 Forest plot of hazard ratios for subgroups stratified by male percentage**

**
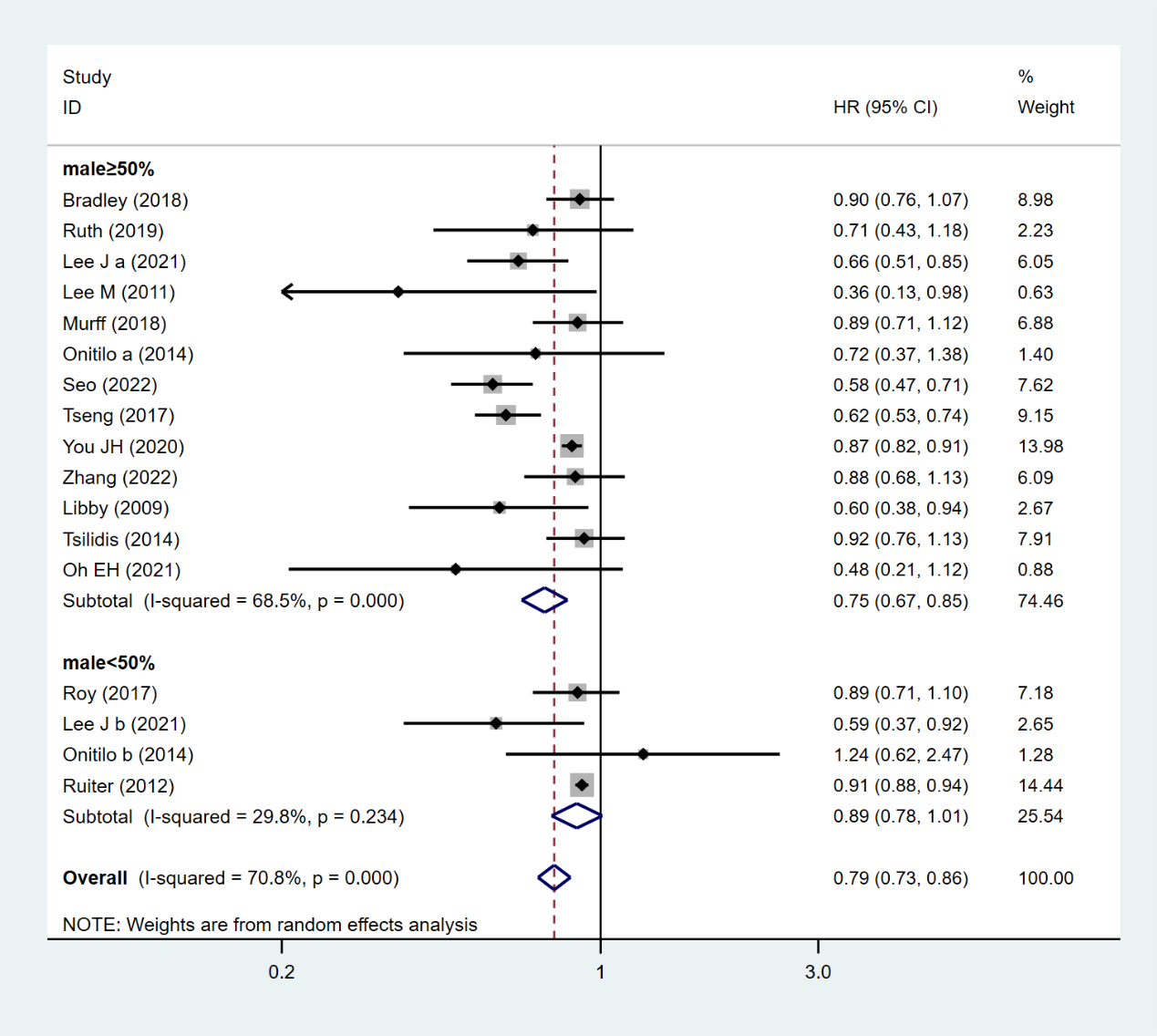
**

**Supplementary Figure 5 Forest plot of hazard ratios for subgroups stratified by mean BMI**


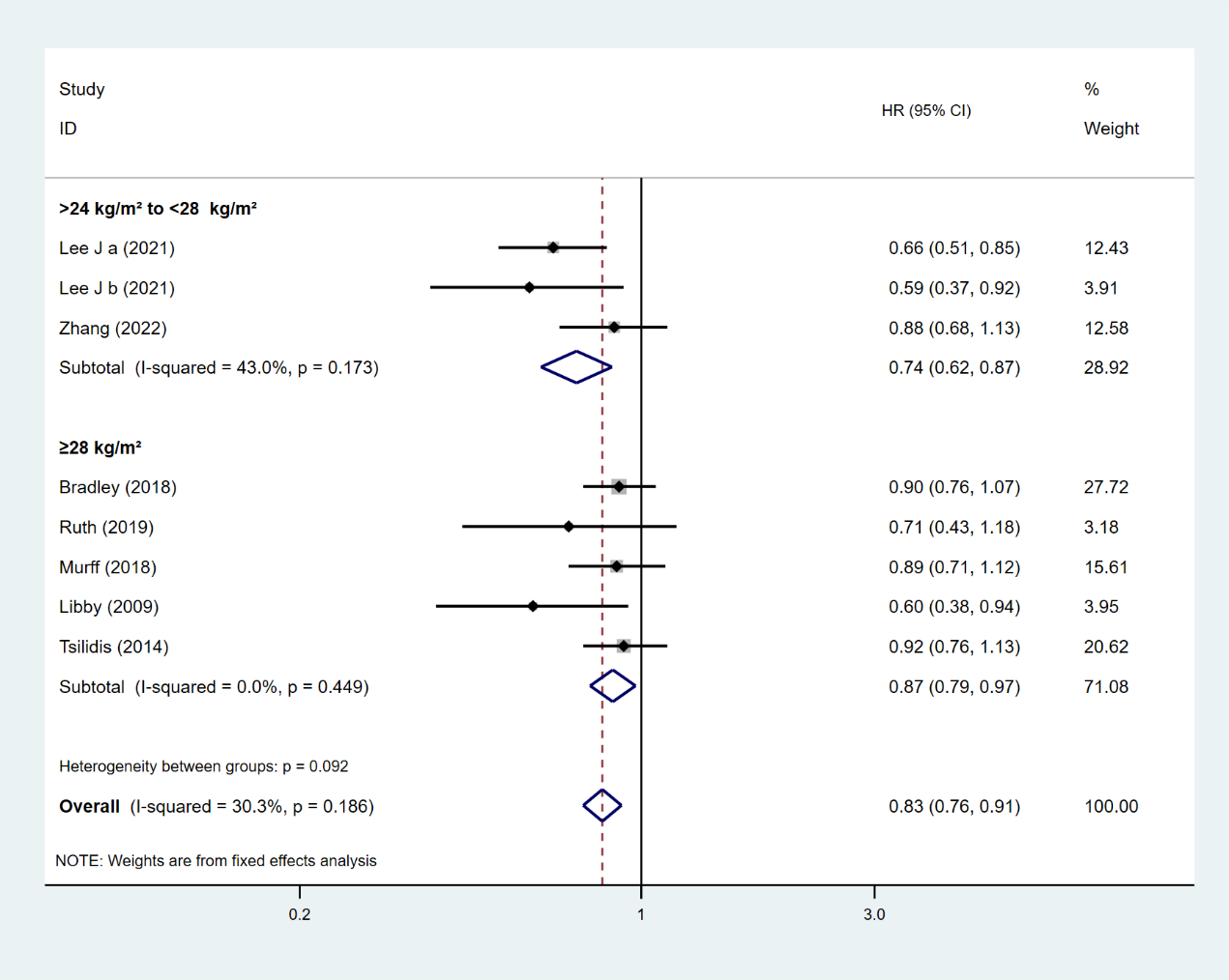


**Supplementary Figure 6 Forest plot of odds ratios for subgroups stratified by outcome**


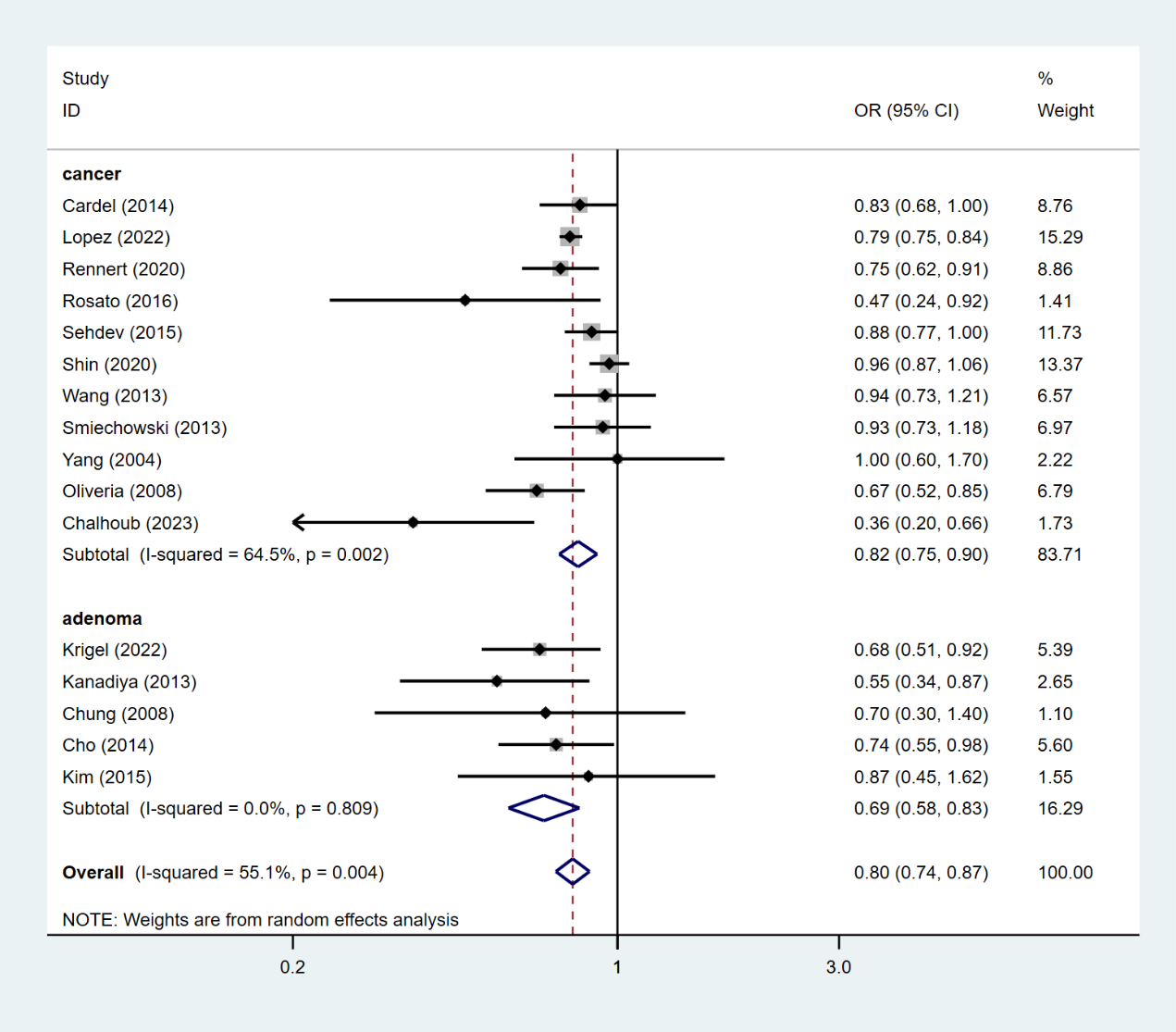


**Supplementary Figure 7** **Forest plot of odds ratios for subgroups stratified by region**


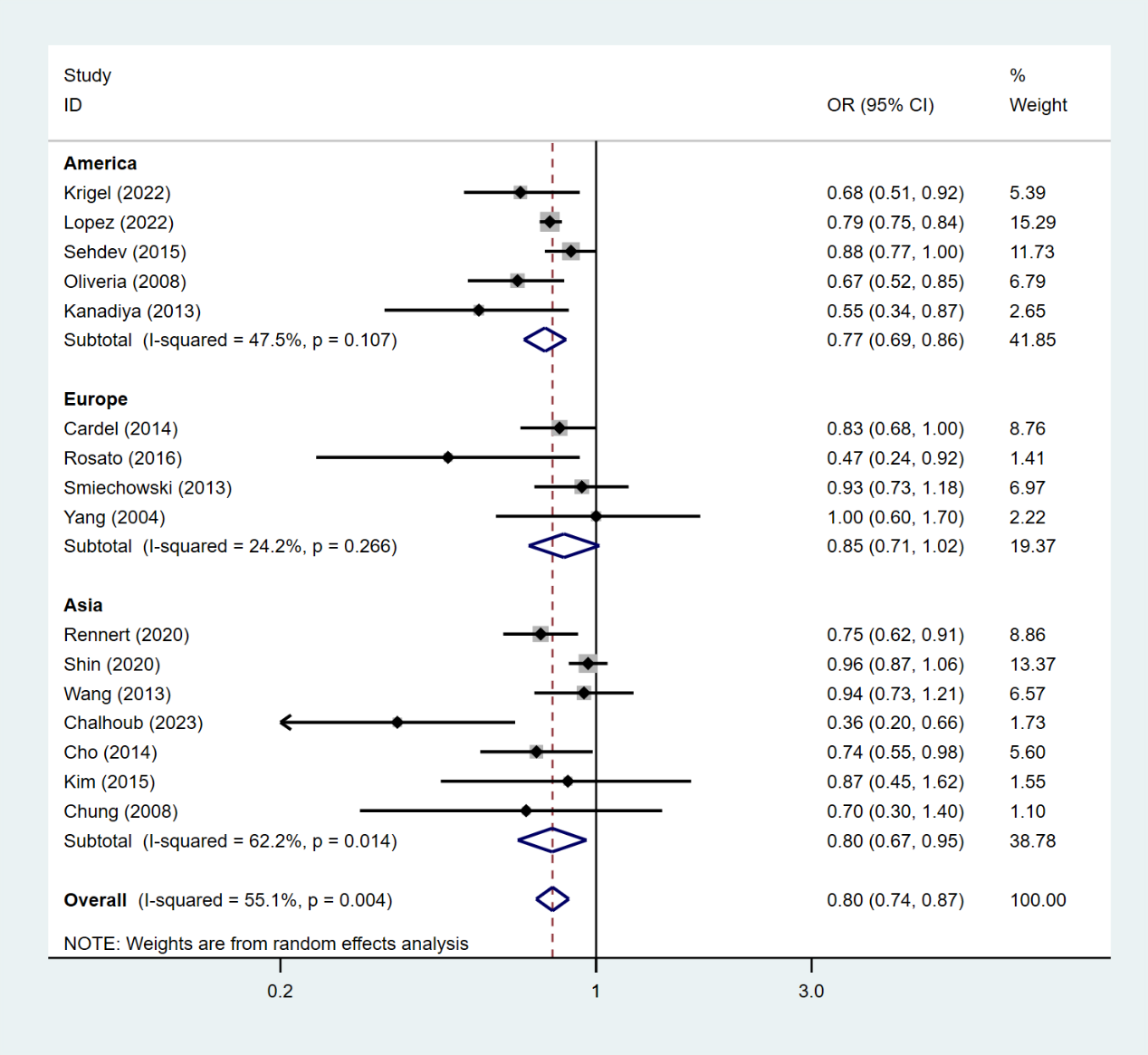


**Supplementary Figure 8 Forest plot of odds ratios for subgroups stratified by mean age**


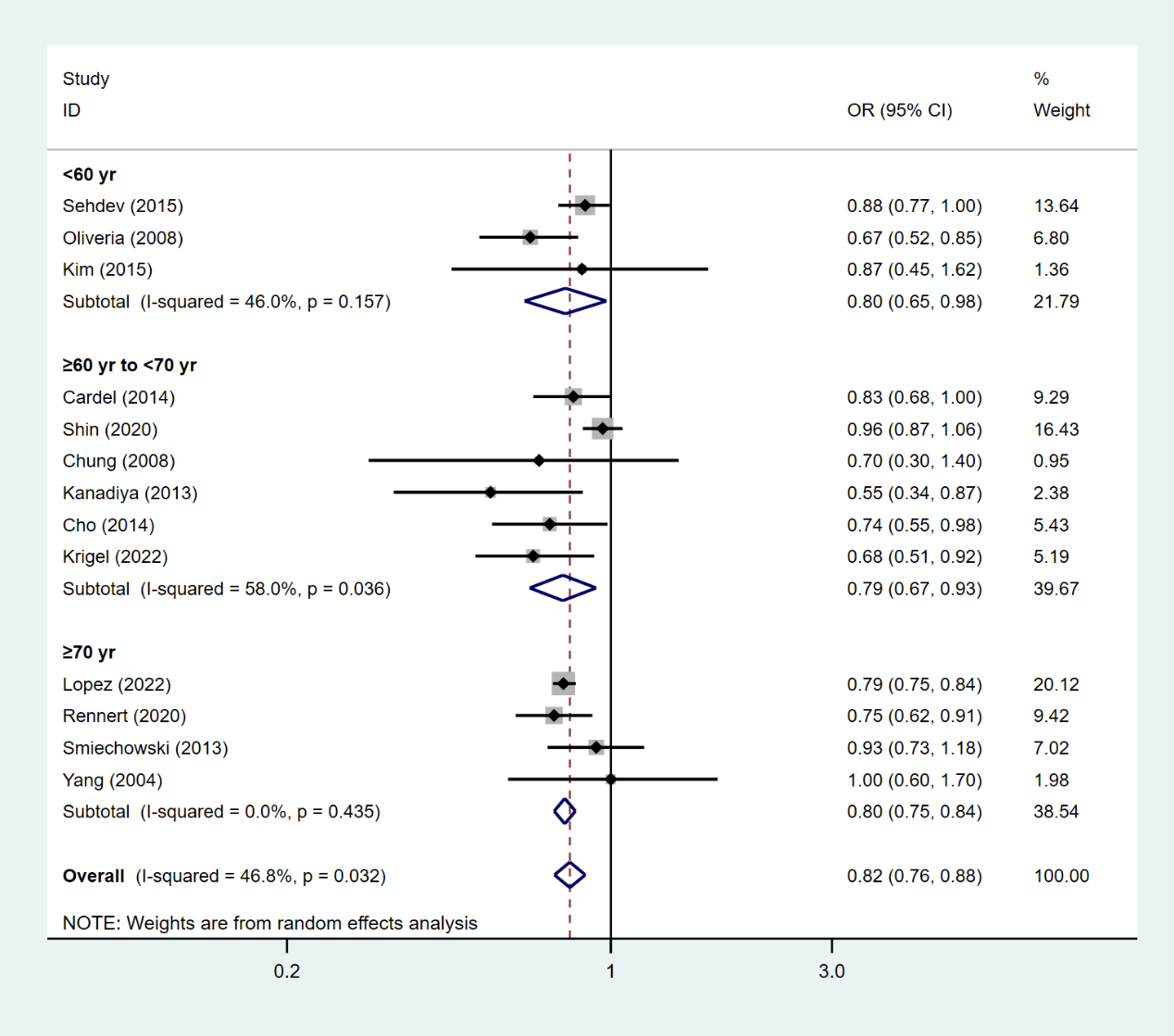


**Supplementary Figure 9** **Forest plot of odds ratios for subgroups stratified by male percentage**


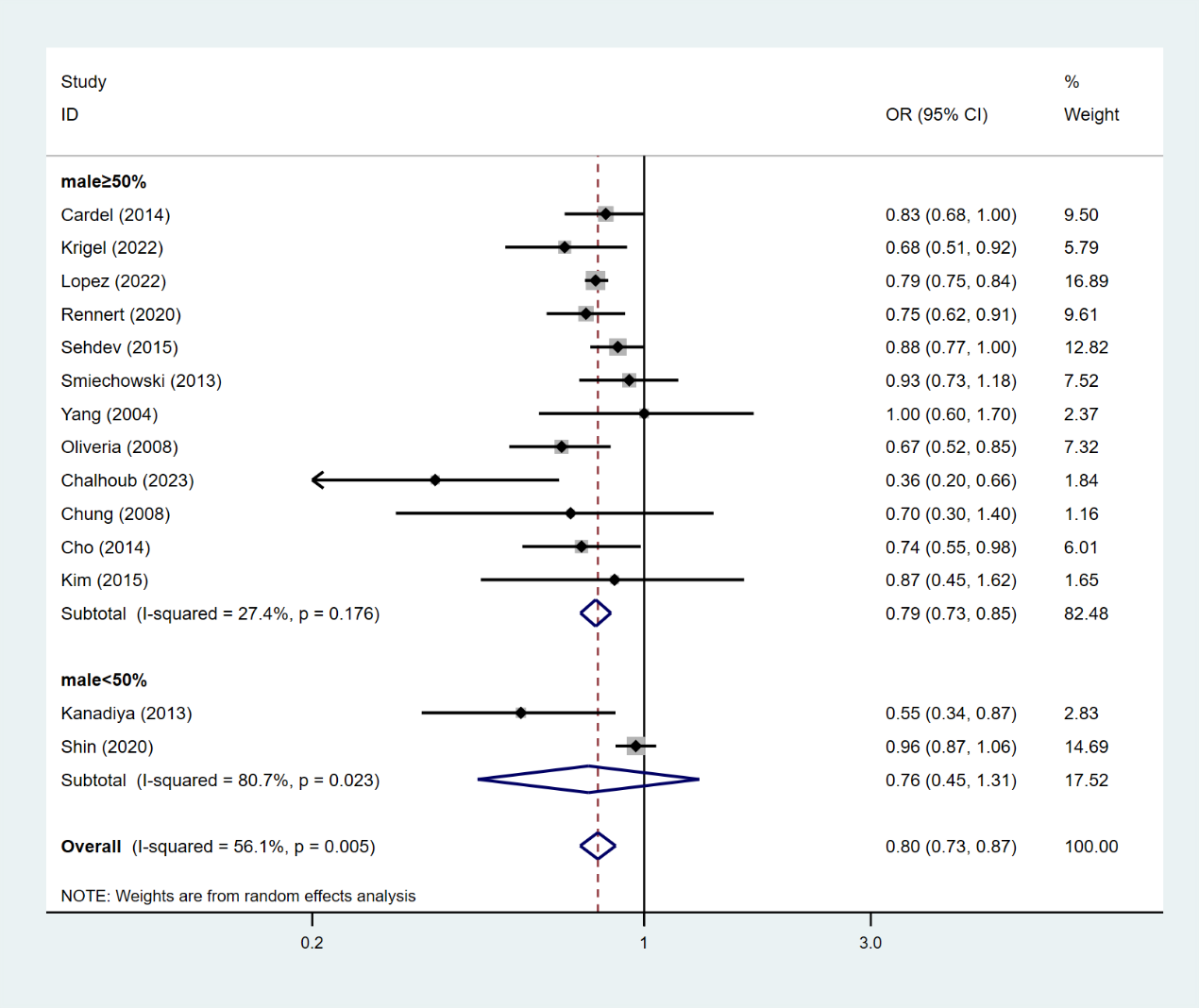


**Supplementary Figure 10 Forest plot of odds ratios for subgroups stratified by mean BMI**


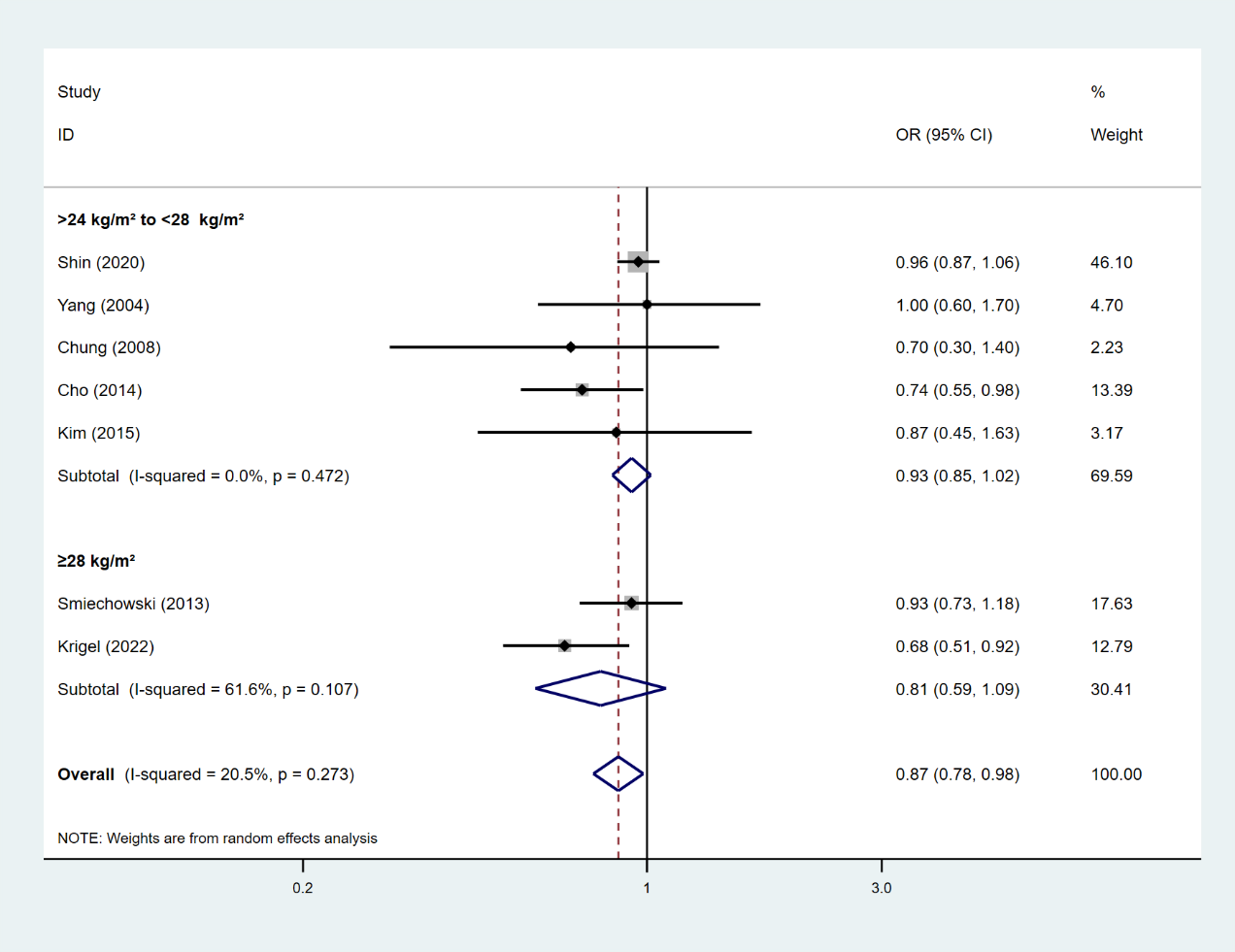


**Supplementary Figure 11 Trim and fill funnel plot of publication bias for studies reporting HR**


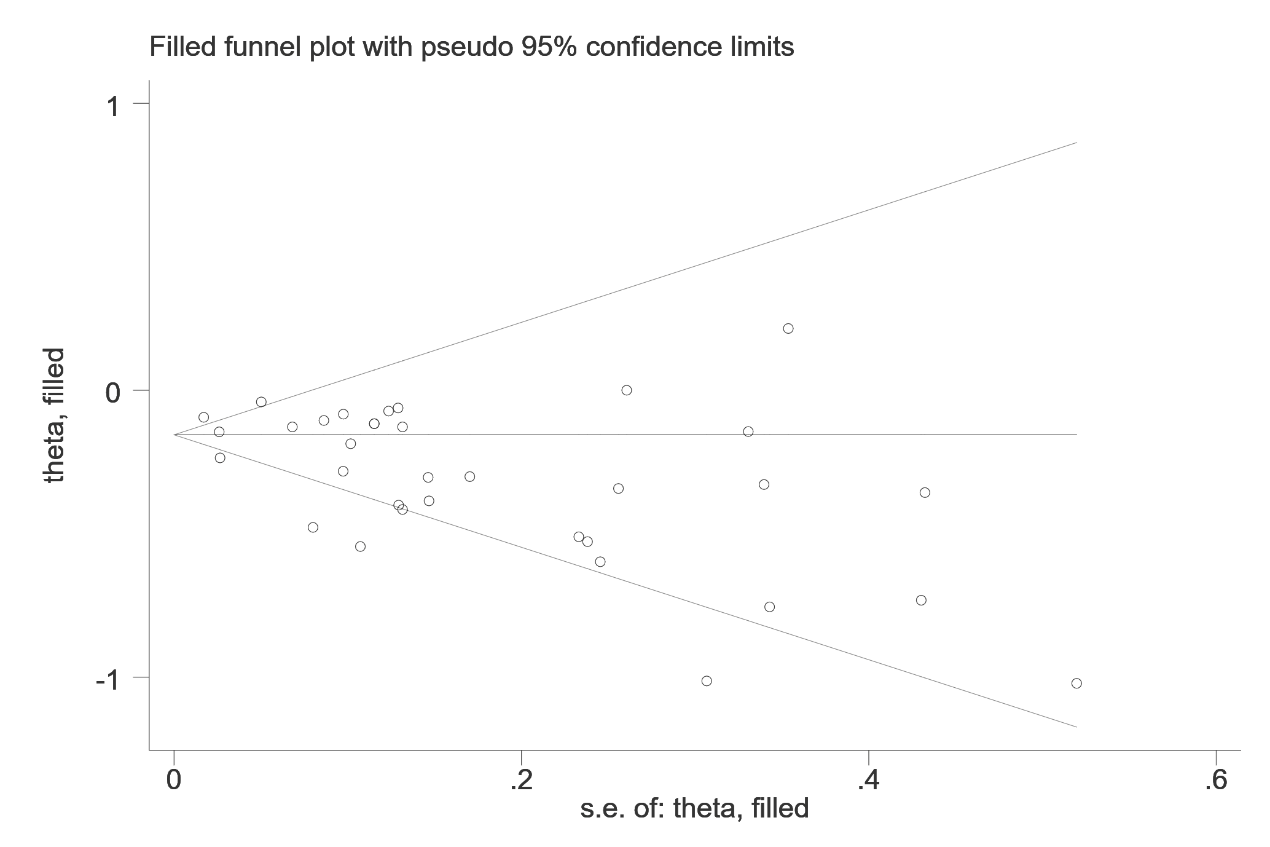


**Supplementary Figure 12** **Hazard ratios of sensitivity analysis for other antidiabetic comparators**


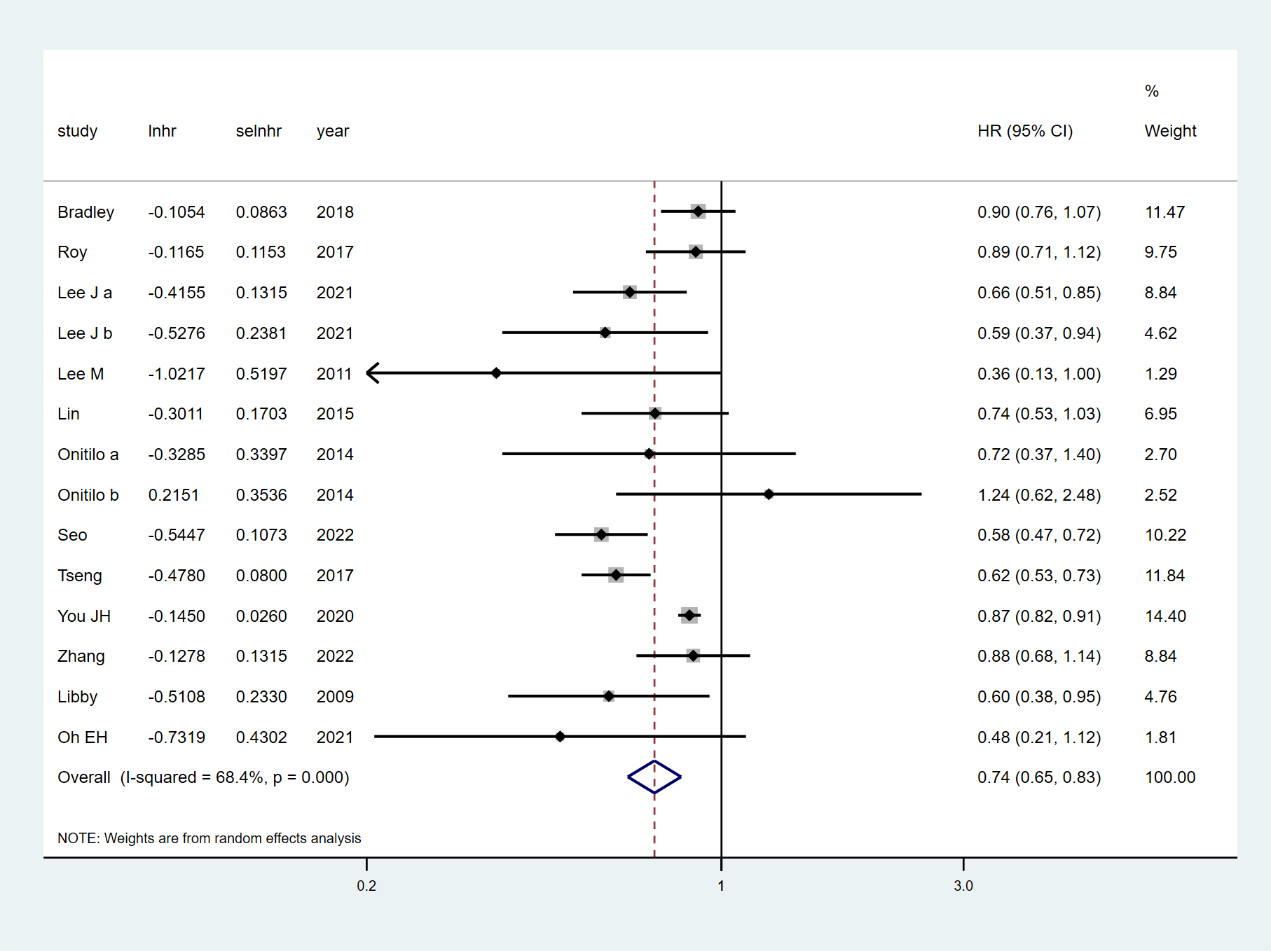


**Supplementary Figure 13 Hazard ratios of sensitivity analysis for high-quality studies**

**
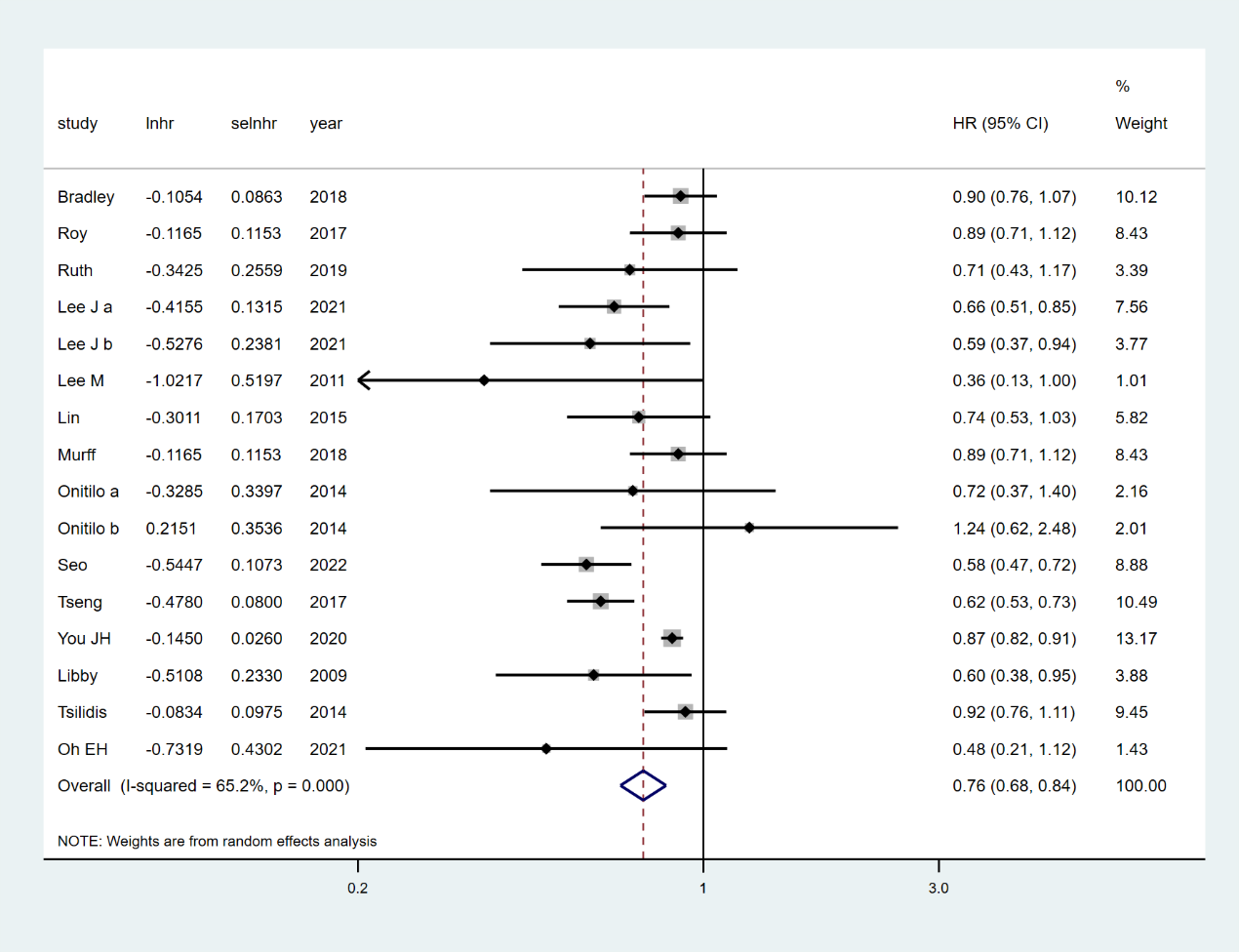
**

**Supplementary Figure 14 Hazard ratios of sensitivity analysis for diabetes-specific population
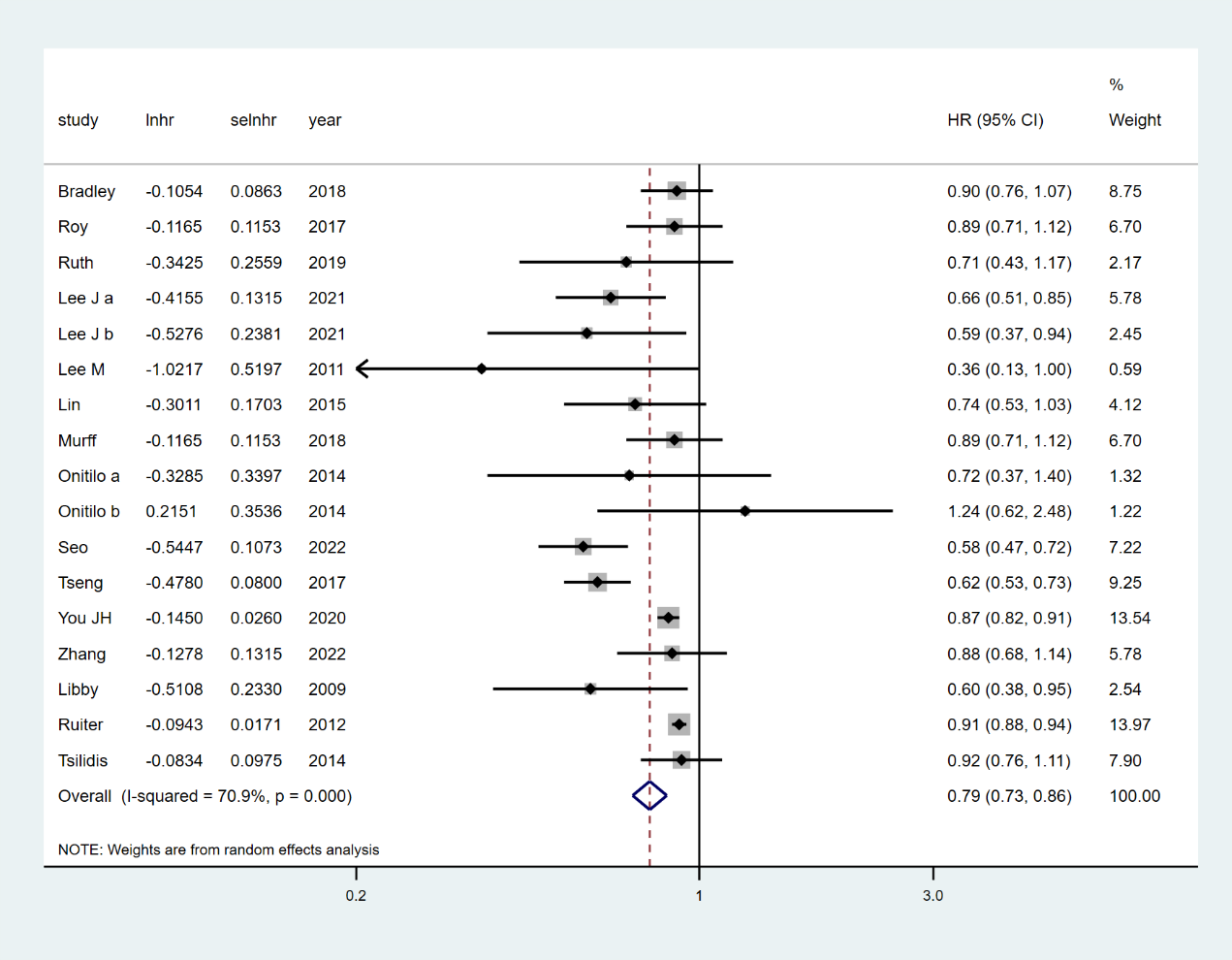
**

**Supplementary Figure 15 Odds ratios of sensitivity analysis for cohort studies only**

**
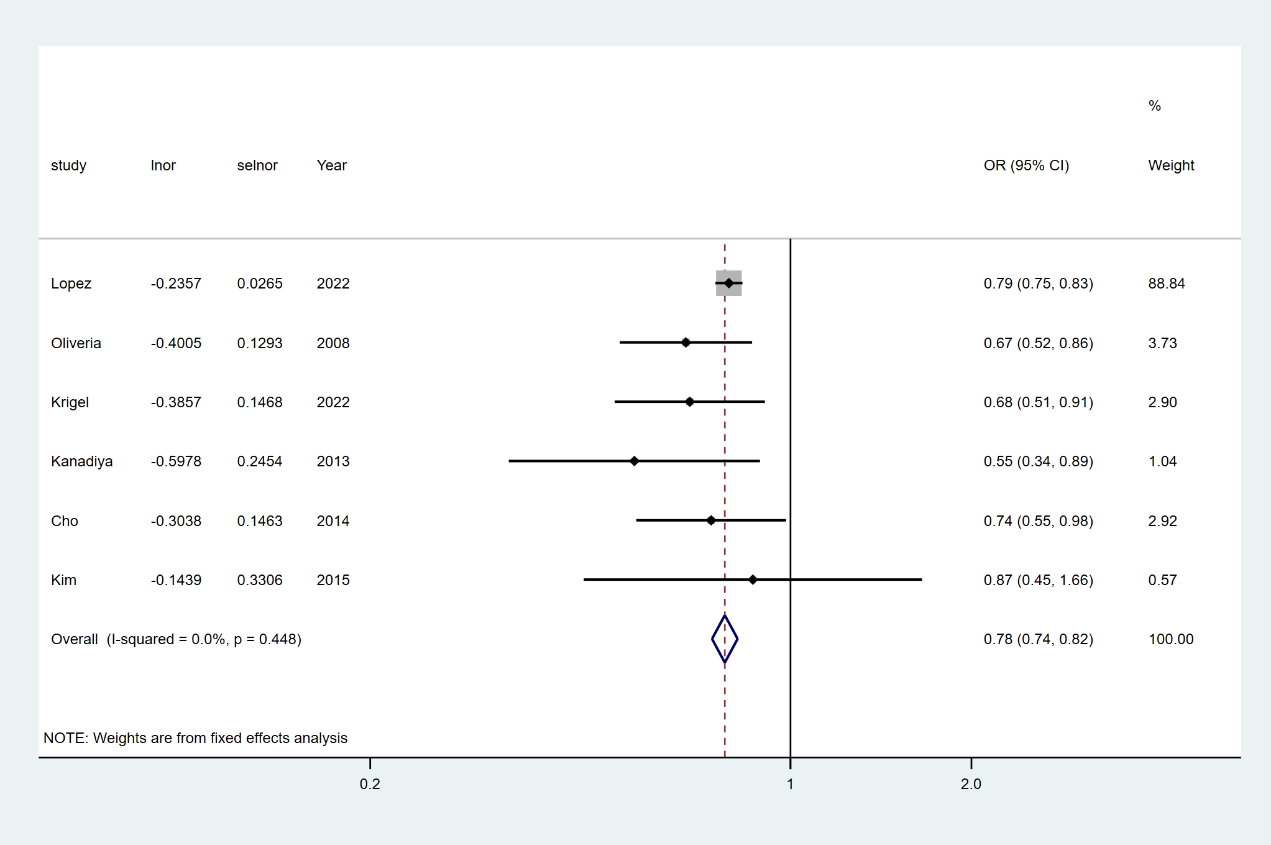
**

**Supplementary Figure 16 Odds ratios of sensitivity analysis for high-quality studies**

**
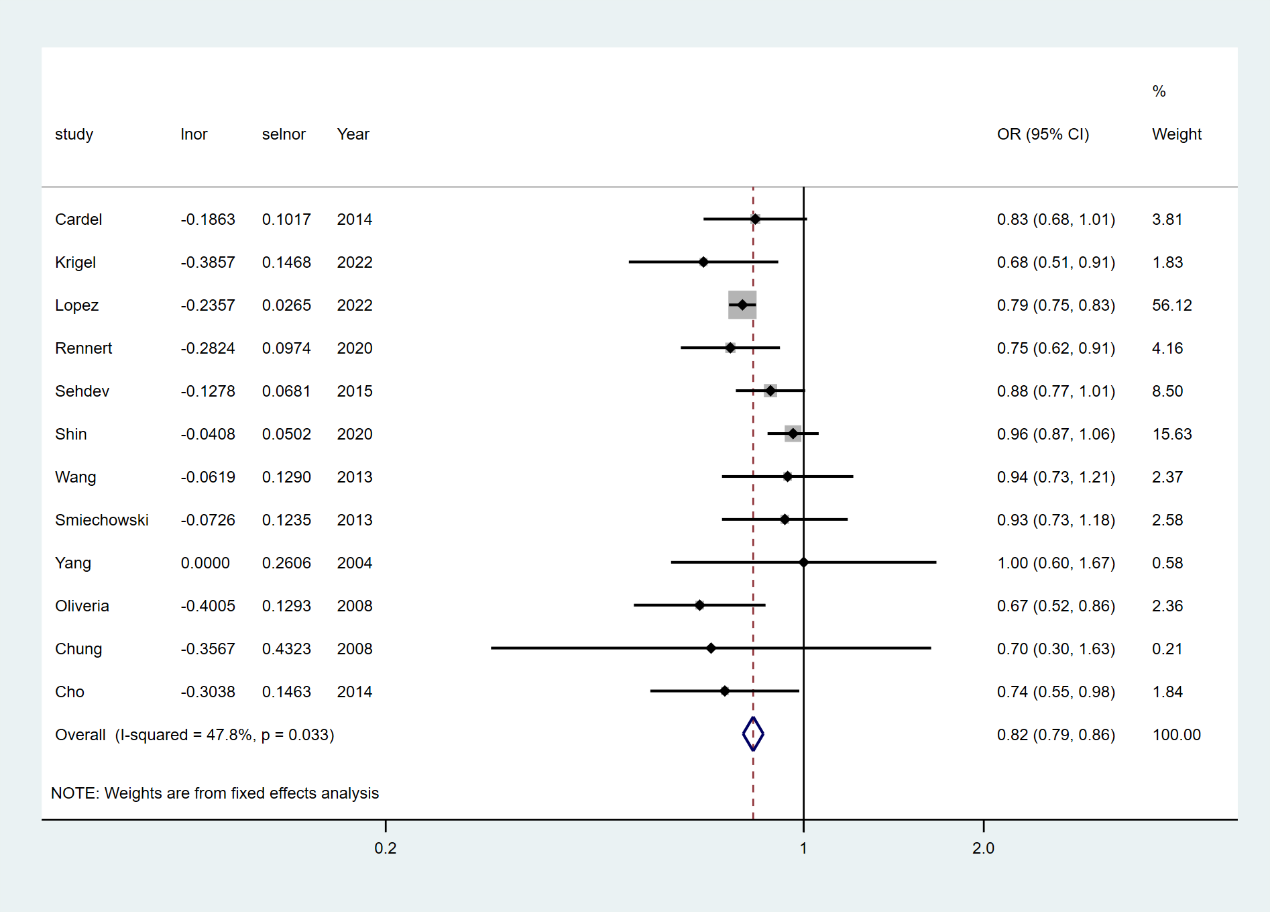
**

**Supplementary Figure 17 Odds ratios of sensitivity analysis for diabetes-specific population**

**
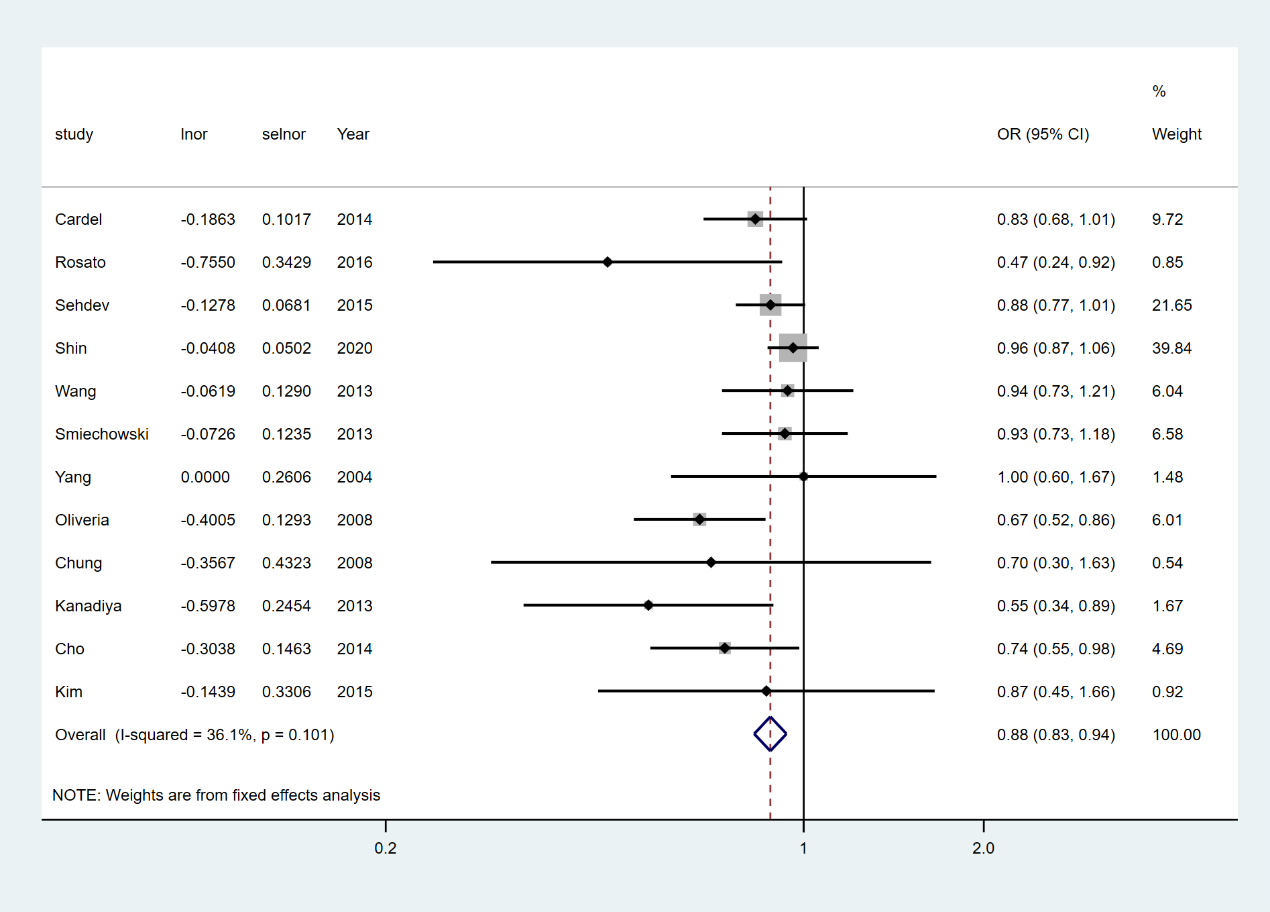
**

**Supplementary Table 6 Dose effect data of studies included in the dose-**

**response analysis**

| First author | Year | Criteria for categories | Categories of metformin intake | OR/RR(95% CI) |
| --- | --- | --- | --- | --- |
| Bodmer | 2012 | metformin duration | 0  1-9 prescriptions  10-29 prescriptions  30-49 prescriptions  ≥50 prescriptions | 1.00  1.05 (0.83－1.33)  1.05 (0.84－1.33)  1.17 (0.88－1.55)  1.43 (1.08－1.90) |
| Bradley | 2018 | cumulative dose | 0  ≤750,000mg  750,001-2,300,000mg  2,300,001-4,930,000mg  >4,930,000mg | 1.00  0.85 (0.68－1.10)  0.99 (0.78－1.24)  0.95 (0.73－1.24)  0.67 (0.45－0.98) |
|  |  | metformin duration | 0  <2.0y  2.0-4.9y  ≥5.0y | 1.00  0.88 (0.70－1.10)  1.02 (0.81－1.27)  0.78 (0.60－1.02) |
| Cardel | 2014 | daily dose | 0  <250mg  250-499mg  500-999mg  1000-1499mg  ≥1500mg | 1.00  1.09 (0.94－1.26)  0.97 (0.81－1.15)  0.98 (0.85－1.13)  0.86 (0.69－1.06)  0.72 (0.50－1.03) |
|  |  | metformin duration | 0  <1 y  1-3 y  3-5 y  5-7 y  7-9 y  >9 y | 1.00  1.09 (0.89－1.32)  0.94 (0.78－1.14)  0.92 (0.75－1.13)  0.82 (0.64－1.04)  0.79 (0.59－1.06)  1.03 (0.71－1.49) |
| Chang | 2018 | daily dose | 0  ≤300mg  300-600mg  600-900mg  >900mg | 1.00  0.70 (0.59－0.84)  0.41 (0.30－0.56)  0.23 (0.13－0.38)  0.15 (0.10－0.24) |
|  |  | metformin duration | 0  <5 y  5-10 y  >10 y | 1.00  0.36 (0.29－0.44)  0.60 (0.49－0.74)  0.59 (0.34－1.00) |
| Roy | 2017 | cumulative dose | 0  <405 g  405–998 g  ≥999 g | 1.00  0.86 (0.65－1.14)  0.89 (0.69－1.15)  0.91 (0.69－1.20) |
| Rennert | 2020 | metformin duration | 0  ≤12 months  13-24 months  25-36 months  37-48 months  49-60 months | 1.00  0.837 (0.699－1.001)  0.855 (0.649－1.126)  0.915 (0.675－1.240)  0.935 (0.685－1.277)  0.850 (0.716－1.010) |
| Rosato | 2016 | metformin duration | 0  1 to <5 y  5 to <10 y  ≥10 y | 1.00  0.74 (0.33－1.66)  0.25 (0.09－0.69)  0.36 (0.15－0.85) |
| Sehdev | 2015 | daily dose | 0  ≤1,000 mg  1,001–1,500 mg  1,501–2,000 mg  >2001 mg | 1.00  1.09 (0.73－1.62)  1.04 (0.79－1.36)  0.89 (0.51－1.55) |
|  |  | metformin duration | 0  ≤123 d  124–240 d  241–313 d  >314 d | 1.00  1.12 (0.81－1.55)  1.04 (0.75－1.44)  0.89 (0.63－1.26) |
| Shin | 2020 | metformin duration | 0  90-180 d  180-360 d  360-730 d  730- d | 1.00  1.00 (0.86－1.17)  0.97 (0.82－1.13)  0.89 (0.77－1.04)  0.97 (0.87－1.09) |
| Tseng | 2017 | metformin duration | 0  <26.8 m  26.8-58.3 m  >58.3 m | 1.00  1.02 (0.81－1.28)  0.70 (0.56－0.89)  0.32 (0.23－0.43) |
| Tseng | 2012 | metformin duration | 0  <1 y  1-3 y  ≥3 y | 1.00  0.876 (0.590－1.301)  0.859 (0.629－1.173)  0.643 (0.490－0.845) |
| Smiechowski | 2013 | metformin duration | 0  <449 d  449–845 d  845–1447 d | 1.00  1.13 (0.85－1.49)  0.82 (0.60－1.12)  0.80 (0.58－1.10) |

**Supplementary Figure 18 Sensitivity analysis for the dose-response relationship**


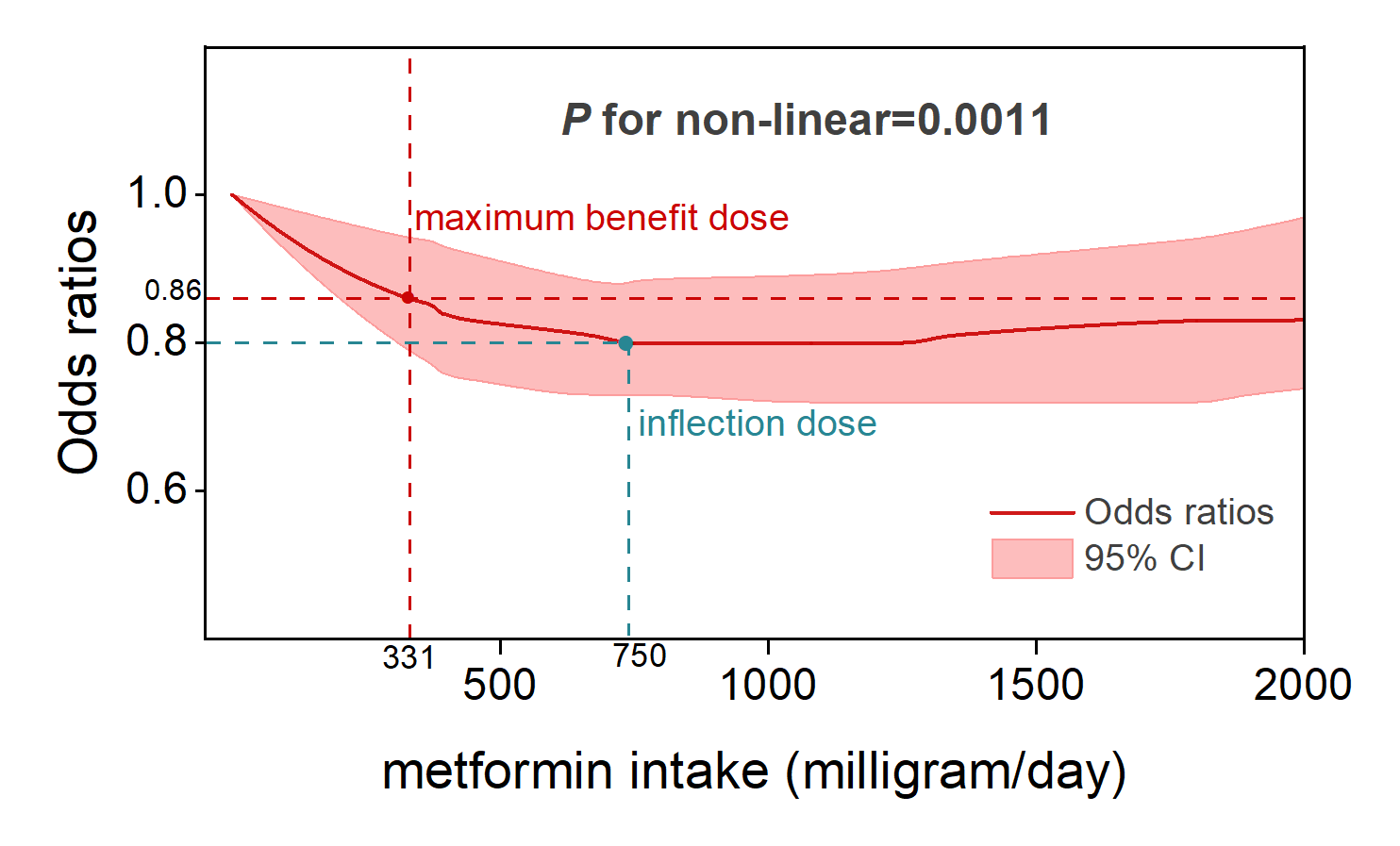

Supplement: Supplementary file 1 [file Supplementaryfile1.docx]
